# A hierarchical Bayesian brain parcellation framework for fusion of functional imaging datasets

**DOI:** 10.1162/imag_a_00408

**Published:** 2025-01-02

**Authors:** Da Zhi, Ladan Shahshahani, Caroline Nettekoven, Ana Luísa Pinho, Danilo Bzdok, Jörn Diedrichsen

**Affiliations:** Western Institute for Neuroscience, Western University, London, Ontario, Canada; Department of Computer Science, Western University, London, Ontario, Canada; Biological & Biomedical Engineering, McGill University, Montreal, QC, Canada; Department of Statistical and Actuarial Sciences, Western University, London, Ontario, Canada

**Keywords:** hierarchical Bayesian model, functional brain parcellation, task-based fMRI

## Abstract

Different task-based and resting-state imaging datasets provide complementary information about the organization of the human brain. Brain parcellations based on single datasets will, therefore, be biased toward the particular type of information present in each dataset. To overcome this limitation, we propose here a hierarchical Bayesian framework that can learn a probabilistic brain parcellation across numerous task-based and resting-state datasets, exploiting their combined strengths. The framework is partitioned into a spatial arrangement model that defines the probability of each voxel belonging to a specific parcel (the probabilistic group atlas), and a set of dataset-specific emission models that define the probability of the observed data given the parcel of the voxel. Using the human cerebellum as an example, we show that the framework optimally combines information from different datasets to achieve a new population-based atlas that outperforms atlases based on single datasets. Furthermore, we demonstrate that using only 10 min of individual data, the framework is able to generate individual brain parcellations that outperform group atlases.

## 1 Introduction

The application of machine learning to functional Magnetic Resonance Imaging (fMRI) data promises better models of brain organization. Brain parcellations are an important type of model, which subdivide the brain into a discrete set of functionally distinct regions. This approach has many practical applications: the defined regions can be used to summarize data, infer functional specialization, or construct network models. A large number of parcellation schemes have been derived from resting-state fMRI datasets (Buckner et al., 2011;Ji et al., 2019;Power et al., 2011;Schaefer et al., 2018;Yeo et al., 2011). Previous studies have shown that functional boundaries detected during resting state are indeed predictive of functional boundaries during task performance (Cole et al., 2014;Laumann et al., 2015;Tavor et al., 2016). However, there is also increasing evidence for systematic differences in the functional organization measured during different tasks and during rest (Cole et al., 2014;Greene et al., 2020;Hasson et al., 2009). It may, therefore, be important to consider multiple types of datasets when deriving brain parcellations.

In recent years, an increasing number of high-quality task-based fMRI datasets that sample a broad range of tasks have become available (King et al., 2019;Nakai & Nishimoto, 2020;Pinho et al., 2018,^2020^). Nonetheless, compared with the large and homogeneous resting-state datasets (Van Essen et al., 2013), task-based datasets usually only contain a small to medium number of individuals and are always limited in the tasks that they cover. It would be, therefore, highly desirable to have a principled way of combining evidence from many datasets into a single model. This is especially important as functional brain organization may not only differ between task and rest, but also between different tasks.

A second important problem is that functional brain organization shows substantial interindividual variations even after anatomical variability is accounted for (Mueller et al., 2013), limiting the usefulness of functional group atlases. One way to address this problem is to use independent individual functional localizer data to derive individual brain parcellation maps (Wang et al., 2015). However, a reliable characterization of brain organization requires an extensive amount of individual functional data (Marek et al., 2018), which in practice is often too costly to acquire.

**Fig. 1.**
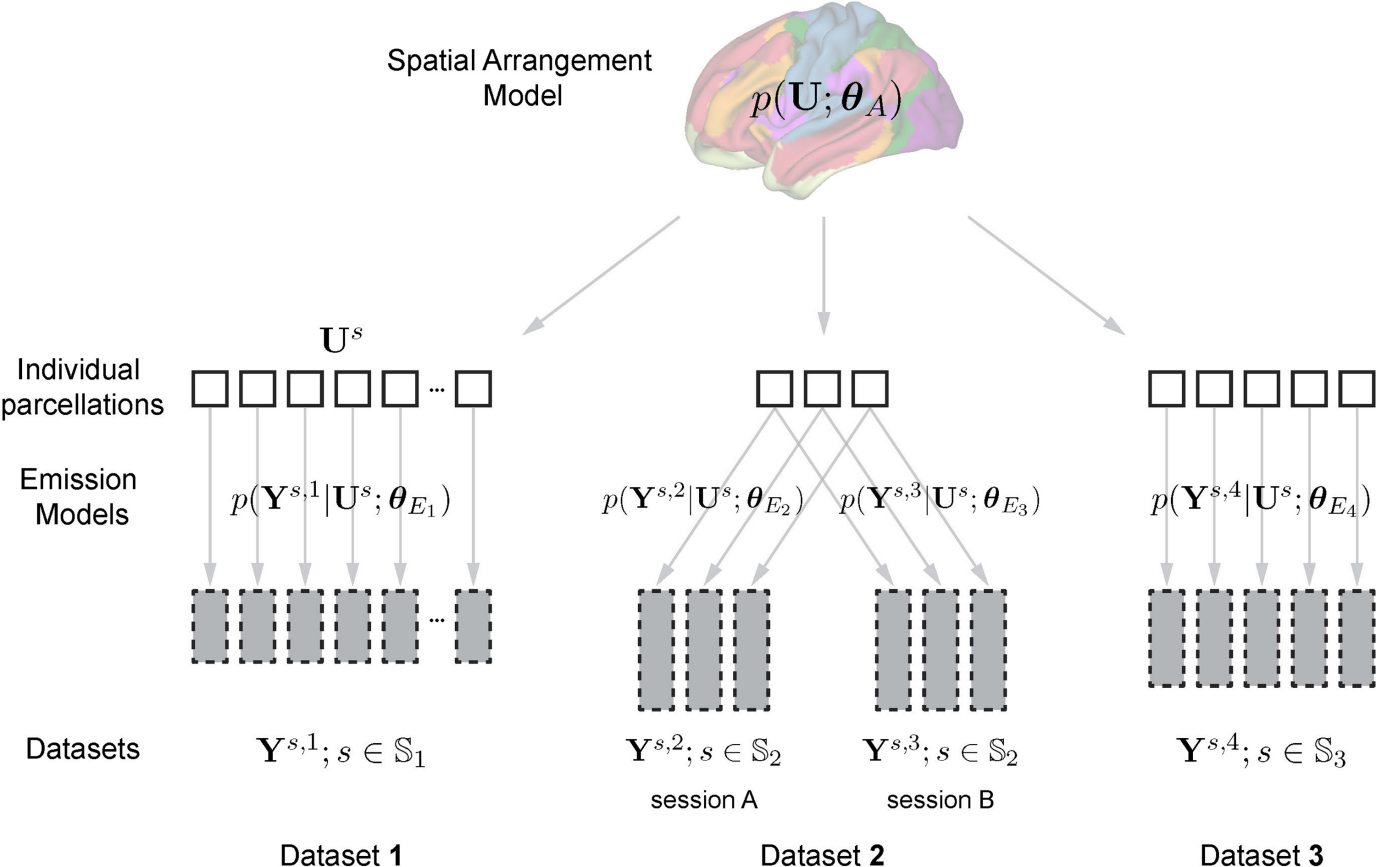
A hierarchical Bayesian parcellation framework for data fusion. Three datasets are shown. Data from each participant and session (${\text{Y}}^{s,i}$) are indicated as a gray box. The height of the box indicates the amount of data. Dataset 2 contains two sessions from the same set of participants ($s\in S_{2}$). The central unknown quantity of the model is the individual brain organization${\text{U}}^{\text{s}}$. The emission models provide the dataset-specific probability of the observed data, given an individual brain organization. The spatial arrangement model provides the population-wide probability of observing a specific brain organization.

In this paper, we present a hierarchical Bayesian parcellation framework (Fig. 1), which addresses both of these problems. The main novelty of our framework, relative to other Bayesian frameworks for brain parcellation (Chong et al., 2017;Kong et al., 2018), is that it is specifically designed to fuse knowledge from different datasets (including task and/or resting-state fMRI data) into a single model. Similar to previous Bayesian frameworks, the model automatically integrates the data from an individual with knowledge from the group atlas to produce an optimal probabilistic parcellation for that individual.

To do this, the central quantities are the*individual parcellations*, which assign each of the possible brain locations in each subjectsto one ofKfunctional regions (here referred to as parcels). The parcel assignments are collected in the matrices${\text{U}}^{s}$, with${\text{U}}_{k,i}^{s}=1$if the$i^{th}$brain location in subjectsis assigned to the$k^{th}$parcel. Linking all individual parcellations is a probabilistic group parcellation, the spatial*arrangement model*,$p({\text{U}}^{s}\;|\theta_{A})$. This model quantifies the probability of how likely a specific brain location belongs to a specific parcel across the studied population. To model different types of fMRI datasets,${\text{Y}}^{s,n}$, recorded in different sessions (n) from different subjects (s), the framework has a collection of dataset-specific*emission models*,$p\left({\text{Y}}^{s,n}|{\text{U}}^{s};\theta_{En}\right)$, the probability of each observed dataset given the individual brain parcellation.

The distributed structure allows the parameters of the model,$\left(\theta_{A},\theta_{E1},...\right)$to be estimated using a message-passing algorithm between the different model components (Section 2.1.4). Once the full model is learned, a new dataset can be added to calculate the expected value of${\text{U}}^{s}$, resulting in a probabilistic parcellation for that individual (seeSection 2.1.1for details).

Starting with a single dataset, we first confirmed that our framework optimally integrates data from a single subject with the group-based arrangement model, resulting in substantially improved individual brain parcellations. We then turn to the main innovation of this paper, namely to explore how to best estimate a single group-based model from multiple datasets. Specifically, we address the question of whether variability in the data needs to be modeled on a session- or even region-specific level. To answer this, we first investigated this issue using simulated data and then tested it on real data.

In this work, we use the cerebellum as an example, as it contains many different distinct functional regions (King et al., 2019), compacted into a small area—making it especially challenging for standard group-based parcellation. We show that parcellations trained on multiple task-based fMRI datasets outperform parcellations trained on single datasets, both in terms of the group map and in their ability to generate accurate individual parcellations on independent data. Finally, we applied the framework to both task-based and resting-state data to test the ability of the framework when datasets are fused from different modalities.

## 2 Methods

### 2.1 A hierarchical Bayesian parcellation framework for data fusion

We introduce a hierarchical Bayesian framework that can be used to learn a probabilistic brain parcellation across multiple fMRI datasets. The framework (Fig. 1) consists of a group-based brain parcellation model (the spatial arrangement model), and a series of dataset-specific emission models. The two parts of the framework are connected by a message-passing and collaborative-learning process, making learning and inference computationally efficient.

The framework is able to learn parcellations from a collection of data${\text{Y}}^{s,n}$recorded from different subjects (s) during different sessions (n).$S_{n}$is the set of subjects for then-th session, and$S:=\left\{S_{1}\cup S_{2}\cup ...\cup S_{n}\right\}$is the entire set of unique subjects. The parcellation model assigns each of thePpossible brain locations in each individualsto one ofKfunctional regions (here referred to as parcels). The parcel assignment for thei-th brain location is denoted in the one-hot encoded vector${\text{u}}_{i}^{s}$, and collected into theK×Pmatrix${\text{U}}^{s}$. This individual brain organization is the central latent variable in the model. The model estimates the expected value,$\langle {\text{U}}^{s}\rangle$, which provides a probabilistic parcellation for that individual—specifically$\langle {\text{u}}_{i,k}^{s}\rangle$is the probability that brain locationiis part of the functional regionk. Note that we use〈⋅〉to denote the expected value throughout.

The arrangement model provides a probabilistic group model of how likely across individuals a specific brain location is assigned to a specific parcel,$p\left(\text{U};\theta_{A}\right)$. This probability depends on a set of (to-be-estimated) parameters of arrangement model$\left(\theta_{A}\right)$. In this paper, we use a spatial arrangement model that estimates these probabilities for each brain location independently (Section 2.1.3), and, therefore, effectively learns a group-based probabilistic brain atlas (seeSection 4for further extensions that also model the spatial dependence).

Each emission model specifies the likelihood of observed data given an individual brain parcellation,$p\left({\text{Y}}^{s,n}|{\text{U}}^{s};\theta_{E}\right)$. For each dataset or session, we introduce a separate emission model with a separate set of emission model parameters ($\theta_{E}$). This allows us to integrate different datasets or sessions with different signal-to-noise levels.

#### 2.1.1 EM algorithm for the hierarchical Bayesian parcellation framework

We used an*Expectation Maximization*(EM) algorithm to optimize the parameters(θ)of the hierarchical Bayesian model. For such models, direct optimization of the log-likelihood,$\text{log }p({\text{Y}}^{s};\theta )$, is not feasible as it would require us to sum over all possible states of the latent variables in the model (here the individual brain parcellations${\text{U}}^{s}$).

The key idea in EM is to introduce a proposal distribution over the latent variablesq(U), and then to optimize the*Evidence Lower Bound*(ELBO) of the model (Blei et al., 2017;Wainwright & Jordan, 2008). The ELBO provides a lower bound to the full likelihood (over all datasets and subjects) that we want to optimize:

$$\sum_{s,n}\langle \text{log }p\left({\text{Y}}^{s,n};\theta \right)\rangle \geq \sum_{s,n}{\langle \text{log }p\left({\text{Y}}^{s,n},{\text{U}}^{s};\theta \right)\rangle }_{q}-{\langle \text{log }q\left({\text{U}}^{s}\right)\rangle }_{q}.$$ (1)

The first term of the ELBO is the expected complete log-likelihoodℒ. Given the model structure, this quantity can be further split into the expected emission log-likelihoods${ℒ}_{En}$for each experiment or session and the expected arrangement log-likelihood${ℒ}_{A}$as

ℒ=∑s,n〈log p(Ys,n,Us;θ)〉q=∑s∈S1〈log p(Ys,1|Us;θE1)〉q+∑s∈S2〈log p(Ys,2|Us;θE2)〉q             +... +∑s〈log p(Us;θA)〉q≜ℒE1+ℒE2 + ... + ℒA, (2)

where the parameters are subdivided into those for the arrangement model,$\theta_{A}$, and those for each of the emission models$\left\{\theta_{E1},\theta_{E2},...\right\}$. This division makes it possible to update the parameters of the arrangement and emission models independently.

In the expectation step, the ELBO is increased by updating the proposal distribution$q({\text{U}}^{s})$to the approximate posterior distribution, given the current set of parameters as

$$\begin{array}{c} q\left({\text{U}}^{s}\right)\;=p\left({\text{U}}^{s}\;|\;{\text{Y}}^{s,1},{\text{Y}}^{s,2},...;\theta \right)\\ \propto p\left({\text{Y}}^{s,1}\;|\;\;{\text{U}}^{s};\theta_{E1}\right)\times p\left({\text{Y}}^{s,2}\;|\;\;{\text{U}}^{s};\theta_{E2}\right)\times ...\;\times p\left({\text{U}}^{s};\theta_{A}\right). \end{array}\;$$ (3)

This step also allows us to calculate the expectation of the latent variables, resulting in an estimate of the individual brain parcellations${\langle {\text{U}}^{s}\rangle }_{q}$. In the maximization step, we update these parameters using these estimated individual brain parcellations. The expectation and maximization steps are then iterated until convergence (Section 2.1.5).

#### 2.1.2 Dataset-specific emission models

One common choice to model fMRI data across different regions is the*Gaussian Mixture Model*(GMM) (Golland et al., 2008). However, the amplitude of fMRI brain signals${\text{y}}_{i}$(whether or not they are normalized by the measurement noise) varies greatly between datasets, participants, and brain locations. That is, two voxels in the same region may have highly correlated signals, but the signal for one voxel may be twice as large as another one. Therefore, an increasing number of modeling approaches for resting-state fMRI data use a mixture of*von Mises-Fisher*(vMF) distributions (Banerjee et al., 2005;Lashkari et al., 2010;Ryali et al., 2013;Schaefer et al., 2018;Yeo et al., 2011). It has been demonstrated that such a directional distribution outperforms the GMM in modeling resting-state fMRI data (Røge et al., 2017). Here, we confirmed that this is also the case for task-based fMRI data: the vMF mixture model performed better than the GMM in the evaluation (SupplementaryFig. S1). We thus used the vMF mixture as our primary emission model.

The probability density function of anN-dimensional (N≥2) vMF distribution for a data point${\text{y}}_{i}$($\left\|{\text{y}}_{i}\right\|\;=1$) is defined as

$$p_{N}\left({\text{y}}_{i}|\text{v},\kappa \right)=c_{N}(\kappa )\cdot \text{exp}\left(\kappa {\text{v}}^{⊺}{\text{y}}_{i}\right),$$ (4)

wherevdenotes the mean direction (‖v‖ =1),κindicates the concentration parameter (κ≥0). The higher the value ofκ, the smaller the variance of the distribution around its mean direction. The normalizing constant$c_{N}(\kappa )$is given by

$$c_{N}(\kappa )=\frac{\kappa^{\frac{N}{2}-1}}{{\left(2\pi \right)}^{\frac{N}{2}}I_{\frac{N}{2}-1}(\kappa )},$$ (5)

where$I_{r}(\cdot )$refers to the modified Bessel function of therorder.

In a vMF mixture model withK-classes, each of the1≤k≤Kparcels is specified with a separate set of parameters$\left\{{\text{v}}_{k},\kappa_{k}\right\}$. Here we assume spatial independence of the measurement noise, such that the data log-likelihood for each subjects, emission modeln, and brain locationican be computed as

$$l_{i,k}^{s,n}=\log p(y_{i}^{s,n}|u_{i}^{s}(k)=1:\theta_{En})=\log c_{N}(\kappa_{k})+\kappa_{k}v_{k}^{\;\;\;\;T}y_{i}^{s}.$$ (6)

We explored three variants of this model: (a)**Type 1**model assumes that the concentration parameter is the same across all sessions and models the concatenated data from all sessions with the same set of subjects in a single emission model; (b)**Type 2**model assumes that different sessions from the same subjects may have different concentration parameters and models each session, therefore, with a different emission model (Fig. 1, Dataset 2). Evidence from different sessions of the same subject is combined during the message passing (eq. 3). The estimated different concentration parameters allow for adaptive weighting of evidence across sessions. The concentration parameter, however, is assumed to be the same across all parcels; (c)**Type 3**model is identical to Type 2 model but employs a different concentration parameter for each session and parcel. In the maximization step, the emission model parameters$\theta_{E}:=\left\{{\text{v}}_{k},\kappa_{k}\right\}$are updated by maximizing the expected emission log-likelihood${ℒ}_{E}$(Supplementary Materials 1).

#### 2.1.3 Spatial arrangement model

The arrangement model aims to provide a probability measure$p\left(\text{U};\theta_{A}\right)$for each unique individual brain parcellation${\text{U}}^{s}$(s∈S) in the studied population. We considered here the most basic architecture for the spatial arrangement model, namely the*independent arrangement model*, where different brain locations are considered to be mutually independent. In this case, the spatial arrangement model simply learns how likely, across all subjects, brain locationibelongs to parcelk, denoted as$p\left({\text{u}}_{i}(k)\right)$. We parameterize this model using a group log-probability parameter$\eta_{i,k}$for each brain locationiand parcelk:

$$p\left({\text{u}}_{i}(k)\right)=\frac{\text{exp}(\eta_{i,k})}{\sum_{j}\text{exp}(\eta_{i,j})}.$$ (7)

#### 2.1.4 Message passing and collaborative learning

Since the full model breaks into different parts (Fig. 1), the learning algorithm can be partitioned into separate E-steps and M-steps for arrangement and emission models (Algorithm 1). The two parts communicate through a*message-passing*process.

In the E-step for the emission model, the data log-likelihood$\ell_{i,k}^{s,n}$(eq. 6) is calculated for each emission model and subject. If there are subjects with more than one session (e.g. Dataset 2 inFig. 1), the data log-likelihoods are then summed for those subjects,

$$\ell_{i,k}^{s}=\sum_{n}\ell_{i,k}^{s,n}.$$ (8)

The combined data log-likelihoods$\ell_{i,k}^{s}$are then collected and passed to the arrangement model. In the E-step for the arrangement model, we calculate the posterior${\langle {\text{u}}_{i}^{s}(k)\rangle }_{q}$for each individual by integrating the data log-likelihoods with the group log-probability parameter of the arrangement model:

**Algorithm 1.** EM algorithm of the fusion framework.

**Input:** K , fMRI data for subject s and experiment/session n $\left\{{\text{Y}}^{s,n},...\right\}$ , initialemission model parameters $\theta_{E}^{\;\;\;\;(0)}$ , initial arrangement model parameters $\eta_{i,k}^{\left(0\right)}$
**Output:** the final estimated parameters $\theta_{\text{E}}^{\left(t\right)}$ , $\eta_{i,k}^{\left(t\right)}$
**1** Initialize: t=0 , $t_{max}\;\;=200$ , Δ=0.01
**2 while** $t\leq t_{max}$ **do**
**3** calculate emission log-likelihoods eq. 6 for each experiment/session:
**4 for** n=1 *to* N **do**
**5** emission E-step for each available subject s in session *n* using eq.S2:
**6** $\ell_{i,k}^{s,n(t)}=log\;p\left({\text{y}}_{i}^{s,n}|{\text{u}}_{i}^{s}(k)=1;\theta_{En}^{\left(t\right)}\right)$
**7 end**
**8** sum emission log-likelihoods across experiments/session for each subject:
**9** $l_{i,k}^{S\;\;\;\;\;(t)}=\sum_{n}l_{i,k}^{s,n(t)}$
**10** arrangement E-step using Supplementary eq.S4:
**11** ${\langle {\text{u}}_{i}^{s}(k)\rangle }_{q}^{\left(t\right)}=\frac{\text{exp}\left(\ell_{i,k}^{s(t)}\;+\;\eta_{i,k}^{\left(t\right)}\right)}{\sum_{j}\text{exp}\left(\ell_{i,j}^{s(t)}\;+\;\eta_{i,j}^{\left(t\right)}\right)}$
**12** calculate expected complete log-likelihood by summing up eq. 10 and eq. 11 :
**13** $\begin{array}{l} {ℒ}^{\left(t\right)}={ℒ}_{A}^{\left(t\right)}+\sum_{n}{ℒ}_{En}^{\left(t\right)}\\ \;\;\;\;\;\;\;=\sum_{s\in S}\sum_{i}\sum_{k}{\langle u_{i}^{s}(k)\rangle }_{q}^{\;\;\;\;\;(t)}\cdot \eta_{i,k}^{\;\;\;\;\;\;(t)}+\sum_{n}\sum_{s\in S_{n}}\sum_{i}\sum_{k}{\langle u_{i}^{s}(k)\rangle }_{q}^{\;\;\;\;\;\;(t)}\cdot l_{i,k}^{s,n(t)} \end{array}$
**14** check converge criterion:
**15 if** t≥1 **and** ${ℒ}^{\left(t\right)}-{ℒ}^{\left(t-1\right)}<\Delta$ **then**
**16 return** $\eta^{\left(t\right)}$ , ${\langle {\text{u}}_{i}^{s}(k)\rangle }_{q}^{\left(t\right)}$
**17 end**
**18** arrangement M-step using Supplementary eq.S6:
**19** $\eta_{i,k}^{\left(t+1\right)}\leftarrow log\sum_{s}{\langle {\text{u}}_{i}^{s}(k)\rangle }_{q}^{\left(t\right)}$
**20 for** n=1 *to* N **do**
**21** emission M-step by eqs.S8 and S9 (Type 1, 2), or S10 and S11 (Type 3)
**22** $\theta_{En}^{\left(t+1\right)}\leftarrow {\text{argmax}}_{\theta_{En}}{ℒ}_{En}^{\left(t\right)}\left(\theta_{En}\right)$
**23 end**
**24** t←t+1
**25 end**

$${\langle u_{i}^{s}(k)\rangle }_{q}=p\left({\text{u}}_{i}^{s}\;=k\;|{\text{y}}_{i}^{s};\theta_{A},\theta_{E}\right)=\frac{\text{exp}\left(\ell_{i,k}^{s}+\eta_{i,k}\right)}{\sum_{j}\text{exp}\left(\ell_{i,j}^{s}+\eta_{i,j}\right)}.$$ (9)

These quantities are then used to calculate the expected emission log-likelihoods${ℒ}_{En}$and the expected arrangement log-likelihood${ℒ}_{A}$. In the case of an independent arrangement model, the expected arrangement log-likelihood${ℒ}_{A}$can be computed in closed form:

$${ℒ}_{A}=\sum_{s\in S}{\langle \text{log }p\left({\text{U}}^{s};\theta_{A}\right)\rangle }_{q}=\sum_{s\in S}\sum_{i}\sum_{k}{\langle {\text{u}}_{i}^{s}(k)\rangle }_{q}\cdot \eta_{i,k}.$$ (10)

Similarly, the expected emission log-likelihood is calculated by multiplying the data log-likelihood ineq. 6with the posterior expectation (eq. 9) and summing these quantities over subjects, brain locations, and parcels:

$${ℒ}_{En}=\sum_{s\in S_{n}}{\langle \text{log }p\left({\text{Y}}^{s,n}|{\text{U}}^{s};\theta_{En}\right)\rangle }_{q}=\sum_{s\in S_{n}}\sum_{i}\sum_{k}{\langle {\text{u}}_{i}^{s}(k)\rangle }_{q}\cdot \ell_{i,k}^{s,n}.$$ (11)

The sum of these expected log-likelihoodsℒ(ineq. 2) is then used as an objective function to track convergence.

Finally, both the parameters of the emission models$\theta_{E_{n}}$and of the arrangement model$\theta_{A}:=\{\eta_{i,k}\}$are updated by maximizing their respective expected log-likelihoods in their M-steps (Supplementary Materials 1).

#### 2.1.5 Initialization and convergence

The initial arrangement model parameters,$\eta_{i,k}^{\left(0\right)},$were randomly drawn from a standard normal distribution. For the initial emission model parameters, the mean direction vectors${\text{v}}_{k}^{\left(0\right)}$were also drawn from a normal distribution and normalized to be unit vectors. The initial concentration parameters$\kappa_{k}^{\left(0\right)}$were randomly drawn from a uniform distribution between 10 to 150, as we wanted to start with a “medium-sized” directional variance.

As for most other complex nonconvex optimization tasks, the issue of local minima and slow convergence also poses problems during learning in our framework. While each emission model quickly learns a set of mean vectors${\text{v}}_{k}$that reasonably approximates the respective dataset, the different parcels are not necessarily aligned across different datasets. This is especially the case when the emission models are randomly and independently initialized. As the arrangement model receives conflicting information from different emission models, it can take a long time to bring the different emission models into alignment.

To solve this problem, it is sufficient to start the algorithm with a single down pass of information from the (randomly initialized) arrangement model to all emission models. That is, during the first iteration of the loop, we skipped the calculation of the emission log-likelihood (lines 3–9) of theAlgorithm 1, setting all$\ell_{i,k}^{s}$to zeros. This “pretraining” helps to align the corresponding parcel assignments across all datasets.

A further technique to improve convergence is to initialize the model from many different random starting points, and only perform a few learning iterations. After this initial phase of learning, we picked the model with the highest expected log-likelihood, and continued learning until the log-likelihood increased less than (Δ=0.01) in a single step. We used 50 initializations, each trained for an initial 30 steps.

Finally, we repeated this entire process a minimum number of 50 times and then continued until the solution with the highest likelihood was found at least 10 times across independent learning runs. This increased our confidence that we had found a solution that could constitute a global maximum.

### 2.2 fMRI datasets

In this project, we considered seven task-based and one resting-state fMRI datasets (seeTable 1), for which the anonymized data were either openly available or provided by the authors. All participants gave informed consent under the experimental protocol reported in the corresponding publication. The data for*Highres-MDTB*(so far unpublished) were acquired under a protocol approved by the Ethics Board of Western University (REF: 107293).

**Table 1.** FMRI datasets used.

Name	Subjects	Unique task conditions	Functional scan time (min/subject)	Voxel size (mm)	Description	Link	Reference
MDTB	24	47	320	3T, 3 mm	Cognitive, motor, perceptual, social	https://openneuro.org/datasets/ds002105/versions/1.1.0	King et al. (2019)
Highres-MDTB	8	9	120	7T, 1.5 mm	Cognitive, motor, perceptual, social	N/A	N/A
Nishimoto	6	103	162	3T, 2 mm	Cognitive, motor, perceptual, social	https://openneuro.org/datasets/ds002306/versions/1.0.3	Nakai and Nishimoto (2020)
IBC	12	208	822	3T, 1.5 mm	Cognitive, motor, perceptual, social	https://openneuro.org/datasets/ds002685/versions/1.3.1	Pinho et al. (2018) ; Pinho et al. (2020) ; Pinho et al. (2024)
WMFS	16	17	65	3T, 3 mm	Motor and working memory task	https://openneuro.org/datasets/ds005148/versions/1.1.0	Shahshahani et al. (2024)
Multi-demand	37	12	100	3T, 2 mm	Executive tasks	N/A	Assem et al. (2024)
Somatotopic	8	6	96	3T, 1.8/2.4 mm	Motor	N/A	Saadon-Grosman et al. (2022)
HCP-unrelated 100	100	None	60	3T, 2 mm	Resting state	https://www.humanconnectome.org/study/hcp-young-adult/data-releases	Van Essen et al. (2013)

All datasets but the last one are task based, together covering a wide range of psychological domains. The last dataset is a subset of the HCP resting-state data.

The task-based datasets are (1) the*Multi-Domain Task Battery*(*MDTB*,King et al., 2019); (2) a high-resolution version of the*MDTB*(*High-res MDTB*; not yet published); (3) the*Nakai & Nishimoto*dataset (Nakai & Nishimoto, 2020); (4) a subset of the*Individual Brain Charting (IBC)*dataset (Pinho et al., 2018,^2020^,^2024^); (5) the*WMFS*dataset (Shahshahani et al., 2024); (6) the*Multi-Demand*dataset (Assem et al., 2024); and (7) the*Somatotopic*dataset (Saadon-Grosman et al., 2022). The first four datasets include a broad range of task conditions from the perceptual, cognitive, motor, and social domains. In the first three datasets, tasks were randomly intermixed in each imaging session. In the*IBC*dataset, individual runs comprised only one task or a few tasks pertaining to a specific cognitive domain. The three last datasets of the list probe a more circumscribed array of functions: the*WMFS*dataset includes verbal working memory tasks (with forward and backward recall) and finger tapping tasks; the*Multi-Demand*dataset includes three executive function tasks (n-back, task-switch, a no-go); and the*Somatotopic*dataset probes foot, hand, glutes, and tongue movements. Lastly, as a resting-state fMRI dataset, we used the*Unrelated 100*subjects, which were made publicly available in the*Human Connectome Project (HCP)*S1200 release (Van Essen et al., 2013).

The task-based datasets were preprocessed using either the*SPM12*software package (Wellcome Department of Imaging Neuroscience, London, UK) or the*FSL*library (Analysis Group, FMRIB, Oxford, UK). For every participant, an anatomical MRI image (T1-weighted MPRAGE, 1 mm isotropic resolution) was acquired in one scanning session. FMRI data (time series acquired with Echo-Planar Imaging, T2*-weighted sequence using Blood-Oxygenation-Level-Dependent contrast) were realigned for head motion within each session, and for different head positions across sessions using the six-parameter rigid body transformation (Friston et al., 1995;Jenkinson et al., 2002). The mean functional image was then coregistered onto the anatomical image, and this transformation was applied to all functional images (Ashburner & Friston, 1997;Greve & Fischl, 2009). No smoothing or group normalization was applied.

A mass-univariate General Linear Model (GLM) was then fitted to the realigned functional data to estimate brain activation per imaging run. Each task condition was modeled as a boxcar function according to the onsets and duration of the given task condition. The corresponding boxcar function was then convolved with the canonical Hemodynamic Response Function (HRF) (Friston, Fletcher, et al., 1998;Friston, Josephs, et al., 1998). The whole-brain mask was applied to the realigned functional volumes to restrict the GLM to voxels inside the brain. Coefficients of the GLM were divided by the root-mean-square error (RMSE) for each voxel, resulting in individual volume-based maps of normalized activity estimates. These functional derivatives, obtained for each task condition and imaging run, served as input to the fMRI dataset integration framework (seeSection 2.3).

The resting-state data were preprocessed using the HCP minimal processing pipeline (Glasser et al., 2013), including structural registration, correction for spatial distortion, head motion, cortical surface mapping, and functional artifact removal (Glasser et al., 2013;Smith et al., 2013). For each imaging run, this resulted in 1200 time points of processed time series for each voxel of the standard MNI152 template (Van Essen et al., 2012) in the cerebellum. To generate the resting-state functional connectivity (rs-FC) fingerprint of the cerebellar voxels from the HCP dataset, a group-level Independent Component Analysis (ICA) was computed on the temporally concatenated functional data for all 100 subjects. We used the group-ICA implemented in FSL’s MELODIC (Jenkinson et al., 2012) with automatic dimensionality estimation, resulting in 1072 group-level components. Sixty-nine signal components were identified from the first 300 ICA components as resting-state networks, using rules and criteria outlined inGriffanti et al. (2017). Lastly, we regressed the 69 group network spatial maps into the subject-and-run-specific cortical time series, resulting in 69 individualized cortical network time courses. The cerebellar rs-FC fingerprints were calculated as Pearson’s correlations of the cerebellar voxel time series with each cortical network time course.

### 2.3 Data structure and anatomical normalization

One important barrier to integrating task contrasts across different fMRI datasets is that these derivative measures are often stored in different atlas spaces (e.g. MNI, fsLR) and with different naming conventions, requiring specialized code for each dataset. To address this problem, we specified a data structure for fMRI derivatives using BIDS-derivative naming convention and file standards (Gorgolewski et al., 2016). For each dataset, we imported the task contrasts (estimates) for each subject, run, and condition that were estimated from minimally preprocessed, non-normalized, and unsmoothed fMRI data (seeSection 2.2). We then developed a toolbox that allowed the automatic and fast extraction of these data in any desired atlas space (surface- or volume-based), at any desired level of smoothing and aggregation across runs. The toolbox is available in a public repository (https://github.com/DiedrichsenLab/Functional_Fusion).

For this project, we focused on the cerebellar data only. Each anatomical image was processed using the SUIT toolbox (Diedrichsen, 2006), which provided cerebellar segmentation and nonlinear normalization into template space. We then extracted the functional data in 3 mm resolution, aligned to the MNI152NLin2009cSym template (Ciric et al., 2022), resulting in 5446 voxel locations for the cerebellum in group space. After extraction, these files were stored using the CIFTI format, resulting in fast and efficient loading times. The sampled functional data of all datasets were smoothed using a Gaussian kernel of 2 mm standard deviation, except the*Somatotopic*dataset that used a 3 mm smoothing kernel. The parcellations were visualized using a surface-based representation of the cerebellum (Diedrichsen & Zotow, 2015).

### 2.4 Synthetic datasets for simulation

To validate the proposed framework, we ran several simulations (Sections 3.2and3.3) on synthetic datasets. To generate the ground-truth individual parcellation maps (${\text{U}}^{s}$), we used a Markov random field of rectangular50×50grid with a 4-neighbor connectivity scheme (seeSupplementary Materials 2). We then generated synthetic functional data${\text{Y}}^{s}$for each participant based on these individual parcellation maps. Rather than using a von Mises–Fisher distribution, we wanted to generate data that had both an amplitude and direction. In addition to the random region-specific mean direction of the response${\text{v}}_{k}$, we, therefore, introduced a nonnegative region-specific signal strength parameter,$\lambda_{k}$. The data for each vertexiwere generated from

$${\text{y}}_{i}\;=\;\;\lambda_{k}{\text{v}}_{k}+\epsilon ,$$ (12)

whereϵwas a normal random vector with variance$\text{I}\cdot \sigma_{k}^{2}$. These parameters allowed us to control the signal and noise levels in each region separately. After normalization of the data to unit length, the generated data conformed approximately to a von Mises–Fisher distribution with mean${\text{v}}_{k}$and concentration$\kappa_{k}\;=\;\;\;\lambda_{k}^{2}/\;\sigma_{k}^{2}$. Ultimately, a synthetic dataset consisting ofNtask observations was generated forPbrain locations andSsubjects.

For the simulation inSections 3.2and3.3, we generated 10 individual parcellations withK=20. For each individual, we then generated two sessions of synthetic data${\text{Y}}^{s,1}$(session 1,N=40tasks),${\text{Y}}^{s,2}$(session 2,N=20tasks), and a test set$Y_{\;\;\;\;\;test}^{s}$(N=120tasks) with equal signal strength$\lambda_{k}\;\;=1.1$for all functional regions. The$\lambda_{k}$was changed depending on specific simulations (seeSections 3.2and3.3).

### 2.5 Evaluation of probabilistic atlases

#### 2.5.1 Group and individual parcellations

To evaluate different probabilistic brain parcellation models, we tested both the performance of the resultant group probability map and the performance of individual parcellations derived from the group map.

To evaluate the group probability map, we split our data into the*atlas training datasets*which were used to estimate the model, and an*evaluation dataset*which was used to calculate the test performance. For the independent arrangement model, the group map could be derived directly from the estimated arrangement parameters, which is thek-long vector of probabilities at each brain location

$$p({\text{u}}_{i})=\text{softmax}(\eta_{i}).$$ (13)

To evaluate individual parcellations (which depend on the probabilistic group map), we further split the evaluation dataset into two parts, an*individual training dataset*and an independent*test dataset*. We first fitted a new emission model to all subjects in the individual training dataset, keeping the parameters of the spatial arrangement model fixed. After convergence, we obtained the individual probabilistic parcellations using a single E-step, which integrates the individual data likelihood with the group probability map. In a vector form,eq. 9can be written as

$$p\left({\text{u}}_{i}^{s}\;|{\text{y}}_{i}^{s};\theta_{A}\right)=\text{softmax}\left(\ell_{i}^{s}+\eta_{i}\right).$$ (14)

For the comparison reported inSection 3.1, we also derived a parcellation only based on data likelihood without taking the group probability into account:

$$p\left({\text{u}}_{i}^{s}\;|{\text{y}}_{i}^{s}\right)=\text{softmax}(\ell_{i}^{s}).$$ (15)

These individual parcellations were then evaluated on the test dataset, which consisted of independent data from the same subjects.

#### 2.5.2 Distance-controlled boundary coefficient (DCBC)

Our main evaluation criterion was the distance-controlled boundary coefficient (DCBC,Zhi et al., 2022) which measures how well a parcellation separates functionally homogeneous regions. For this, the probabilistic (group or individual) parcellation was first transformed into a hard parcellation by assigning each brain location to the parcel with the highest probability. Similar to other clustering evaluation criteria, such as homogeneity and Silhouette coefficients (Gordon et al., 2016;Rousseeuw, 1987), the DCBC then compares the similarity of within-parcel with the between-parcel pairs of brain locations. Given the intrinsic smoothness of brain functional data, traditional metrics are biased in favor of finer parcellations, such that they do not allow for comparisons of parcellations with different number of parcels. The DCBC method solves this problem by binning all vertex pairs based on their spatial distance and only comparing Pearson’s correlation for within-parcel pairs and between-parcel pairs with the same distance. The overall DCBC value is calculated as the average correlation difference, weighted by the inverse of the variance of the correlation difference (estimated based on the number of within- and between-voxel pairs in each distance bin). The spatial distance was calculated as the Euclidean distance between the center of each voxel pair in the atlas volume space. The underlying functional profiles for calculating the correlations of voxel pairs were the normalized activity estimates for the task-based dataset (see above). A higher DCBC value of a parcellation indicates a better prediction of the functional boundaries in the test dataset.

Overall, the group DCBC evaluates the winner-take-all version of the group map, whereas the DCBC for individual parcellations evaluates the actual parcel**probabilities**of the group map, as these are essential in correctly determining the individual parcellations.

### 2.6 Computational setup

Model training and evaluations were performed on either an NVIDIA 1080Ti GPU with Python 3, CUDA 11.3, and PyTorch 1.10.2 or on NVIDIA GRID A100-10C GPU with Python 3, CUDA 11.6, and PyTorch 1.13.1. For the fMRI datasets, all data were preprocessed and extracted on an Intel i7-8700 CPU with NumPy 1.24.0, NiBabel 4.0.2, neuroimagingtools 0.5.0. Other detailed requirements and parameters used for the data processing pipeline are available in the respective repositories (seeData and Code Availability Section).

## 3 Results

### 3.1 Individual parcellations

Given the substantial interindividual functional variability, it is often desirable to derive parcellations for single subjects. Similar to a previous Bayesian brain parcellation model (Kong et al., 2018), our framework explicitly models the interindividual variability in brain organization, and can, therefore, be used to improve individual parcellations. Specifically, our model does offer not only a parcellation based on the learned group parameters,$p\left({\text{U}}^{s}\;|\theta_{A}\right)$, or based on a subject-specific data$p\left({\text{U}}^{s}|\;{\text{Y}}^{s}\right)$, but also an optimal integration of individual data with the group-level probability map (Section 2.5.1). This can be especially useful if only restricted individual data are available.

We first sought to determine how much improvement this integrated estimate offers. For this, we first trained a group parcellation (17 parcels) on the cerebellar data of the first task set of the multidomain task battery dataset (*MDTB*,King et al., 2019). Individual parcellations were then derived using between 1 and 16 imaging runs (10–160 min) of individual training data (eq. 15). We compared the performance of these “data-only” parcellations with the group parcellation (eq. 13), and with the Bayesian integration of the group map with the individual data (eq. 14). We evaluated all parcellations on the second task set of the MDTB, an independent dataset with separate tasks acquired from the same participants. We determined how well the parcellations isolated separated functional homogeneous regions using the DCBC (Section 2.5.2).

**Fig. 2.**
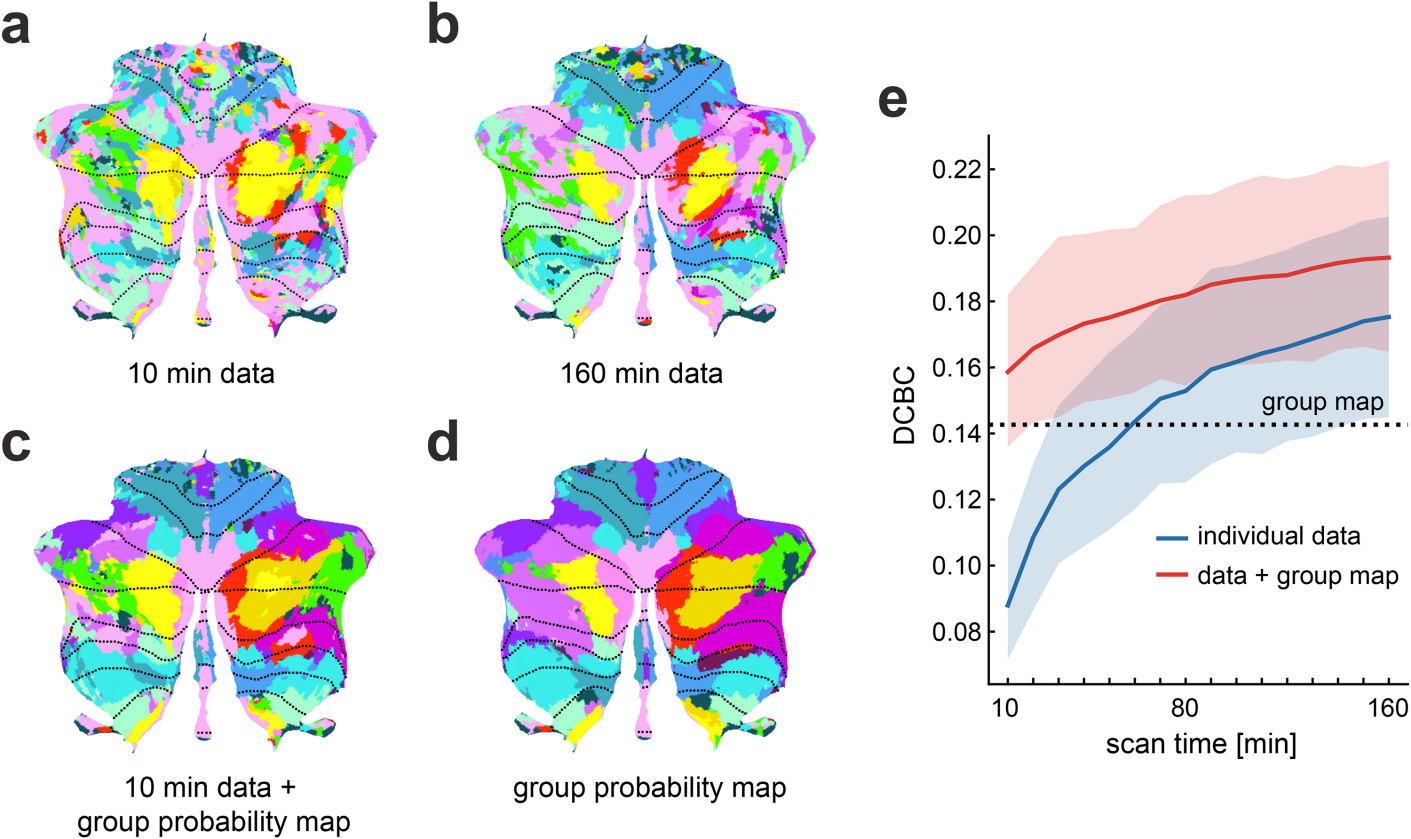
Individual parcellations from our Hierarchical Bayesian framework outperform both purely data-driven parcellations and the group map. (a) An estimated individual parcellation based on 10 min (1 run) of imaging data, using only the individual data. (b) An estimated individual parcellation of the same subject based on 160 min (16 runs), using only the individual data. (c) The integrated individual parcellation estimate using 10 min of individual data and the group probability map. (d) The group probability map. For visualization, all probabilistic maps are converted to hard parcellations. (e) The DCBC value (higher = better) of the parcellations tested on the independent second session of the*MDTB*dataset. Individual parcellation were estimated either using only the individual data (blue curve) or using the posterior probability that integrates individual data and the learned group probability map (red curve). The x-axis indicates the length of the imaging time series (10 min = 1 run) used in estimation. Error bars represent the standard error of the mean across all 24 subjects.

The individual parcellations based on 10 min of imaging data (without using the group probability map,Fig. 2a) performed generally poorly, with an average DCBC of 0.088 (Standard Error of the Mean, SEM = 0.009). Indeed, the individual parcellations performed worse than the group map$t_{23}=-7.786,\;\;p=6.815\times 10^{-8}$(Fig. 2d, dashed line inFig. 2e). As expected, the individual parcellations improved continuously when using more data (Fig. 2b), reaching an average DCBC value of 0.175 (SEM = 0.016) for 160 min of data, ultimately outperforming the group map ($t_{23}=3.286,p=0.003$). This indicates that with sufficient data, we can capture replicable differences in brain organization across individuals. Individual parcellations can capture these differences, leading to significantly better prediction performance than a group probability map on independent test data.

Although individual parcellations were superior to the group map using more data (blue line inFig. 2e), in our study, 110 min of individual imaging data were required to obtain a brain parcellation that was significantly better than the group probability map ($t_{23}=2.190,p=0.039$). At 60 min of imaging, the individual parcellation map was only just about as predictive as the group probability map.

These results confirm that a substantial amount of data is required to obtain a reliable individual parcellation (Marek et al., 2018). However, acquiring this amount of individual data for functional localization is rarely feasible in basic and clinical functional imaging studies. Our framework automatically integrates the individual data with the group probability map, leading to dramatically improved performance. Using only 10 min of individual data, this integrated estimate had a significantly higher DCBC than the group probability map ($t_{23}=3.123,p=0.005$), and performed roughly as well as 100 min of individual imaging data only.

The resultant individual parcellation map (Fig. 2c) constitutes an optimal fusion of the individual data and the knowledge learned from the entire group. Even when 160 min of individual data were available, the integration with the group map led to a significant improvement relative to using only the individual data ($t_{23}=5.562,p=1.171\times 10^{-5}$). Another advantage of the integration of group and individual data is that it naturally deals with missing individual brain data (see SupplementaryFig. S2a, bfor visualization of individual parcellations). For brain locations where the individual data are missing, the parcellation will simply be determined by the group probability map.

To further test the ability of our framework in generating individual parcellations, we replicated this analysis using a different evaluation criterion (prediction error for unseen activity patterns, seeSupplementary Material 3) and a different atlas in our companion paper (Nettekoven et al., 2024). We also performed a reproducibility test of the individual parcellations across different sessions and task sets. The result (SupplementaryFig. S2d) shows that the individual parcellations exhibit significantly higher within-subject similarity than between-subjects similarity, suggesting our framework is able to generate reliable individual parcellations. Altogether, these analyses show that our framework is able to generate improved individual parcellations compared with the group-averaged models, or individual data-only models (Thirion et al., 2024).

### 3.2 Dataset-specific emission models optimally capture differences in measurement noise

**Fig. 3.**
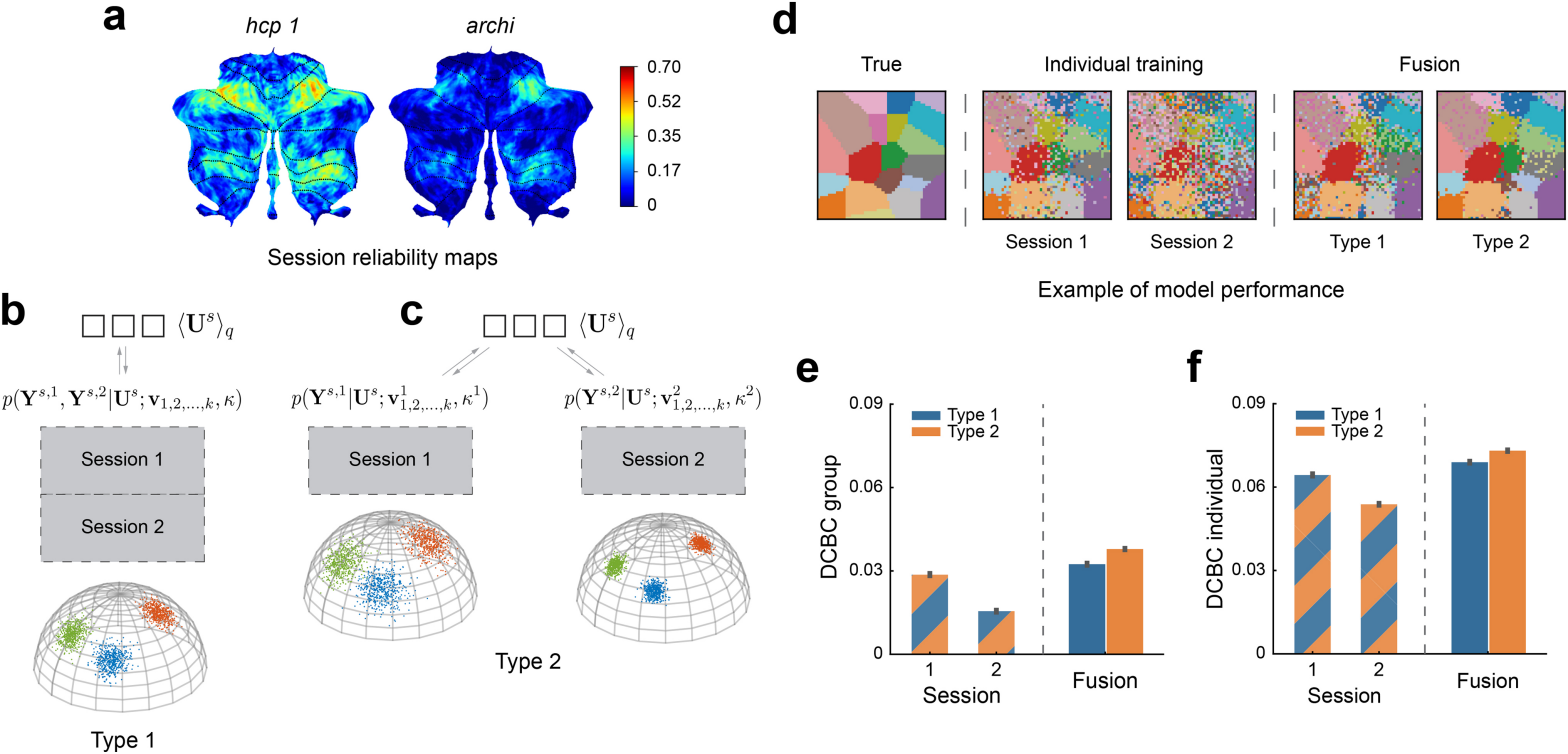
Simulations of data fusion using two synthetic imaging sessions with similar task activation. (a) The split-half reliability of the functional profiles for two imaging sessions from the IBC dataset with similar task sets (*hcp1*and*archi*). The split-half correlation is computed for each voxel within each subject and then averaged across participants. (b) Type 1 model: sessions are concatenated and will be learned in a single emission model with a single concentration parameter. (c) Type 2 model: sessions are separated and modeled using two emission models with separate concentration parameters. (d) Reconstruction of the true parcellation map using synthetic data, using Session 1 or 2 alone vs. the fusion of both sessions using either model Type 1 or 2. (e) The mean DCBC value of the group map learned from Session 1 or 2 alone or from the fusion of both sessions (using model Type 1 or Type 2). (f) The mean DCBC value of individual parcellations. Error bars indicate SEM (standard error of the mean) across 100 simulations.

The main innovation of our framework is that it can integrate different task-based datasets. Different imaging datasets, however, often show very different signal-to-noise ratios. This is the case across datasets, but also across different sessions within a single dataset. For instance, two different imaging sessions of the IBC dataset (Fig. 3a,Section 2.2) show different levels of within-subject reliability. Our framework can potentially deal with these differences by estimating separate concentration parameters for different sessions, such that each session is weighted according to its signal-to-noise ratio.

To evaluate the effectiveness of this approach, we compared different versions of our model. In Type 1 model, different sessions from a single individual were concatenated and modeled with a single emission model and concentration parameter (Fig. 3b). In this scenario, however, the second, noisier session may make the integrated model worse than the first session alone. Therefore, in a different version of the model (Type 2), each imaging session was modeled with a separate emission model. This allowed differences in variability to be captured by a session-specific concentration parameter (e.g.$\kappa^{1}$for session 1 and$\kappa^{2}$for session 2 inFig. 3c). As long as theκ′s are estimated accurately, the subsequent Bayesian integration will ensure the optimal weighting across the different sessions. Therefore, even the addition of a low-quality dataset should never lead to decreases in the quality of the integrated model.

To test this idea, we generated two synthetic datasets (sessions) sampled from the same set of subjects with similar task activation but different overall noise variances (Section 2.4). The measurement noise was set to$\sigma_{k}^{2}=0.5$for synthetic session 1 and to$\sigma_{k}^{2}=0.8$for session 2. We then learned group and individual parcellations using Type 1 or Type 2 models, either using each session alone or fusing both sessions. We then tested the performance of all models on an independent simulated test set (Section 2.4), repeating the simulation 100 times.

Visual inspection of the group parcellations (Fig. 3d) suggests that the group map trained on session 1 alone approximates the true map more accurately than using session 2. The fusion of both sessions improved the group reconstruction, especially when using separate emission models (Type 2). We evaluated the parcellation performances quantitatively using the DCBC measure on the test set (Fig. 3e,3f). The fused parcellation learned by Type 1 fusion model performed better than the parcellation trained on session 1 alone by 0.004 (SD =$3.752\times 10^{-3}$) for the group DCBC and by 0.005 (SD=$3.781\times 10^{-3}$) for the individual DCBC. The parcellation derived from Type 2 model outperformed Type 1 by 0.005 (SD =$4.006\times 10^{-3}$) for the group DCBC and 0.004 (SD=$4.666\times 10^{-3}$) for the individual DCBC. Similar results are obtained using the expected reconstruction error of the true parcellation maps (see SupplementaryFig. S4a). These simulations demonstrate that session-specific emission models allow for better fusion when the signal-to-noise level differs across sessions or datasets.

### 3.3 Region-specific concentration parameters further improve fusion parcellation

**Fig. 4.**
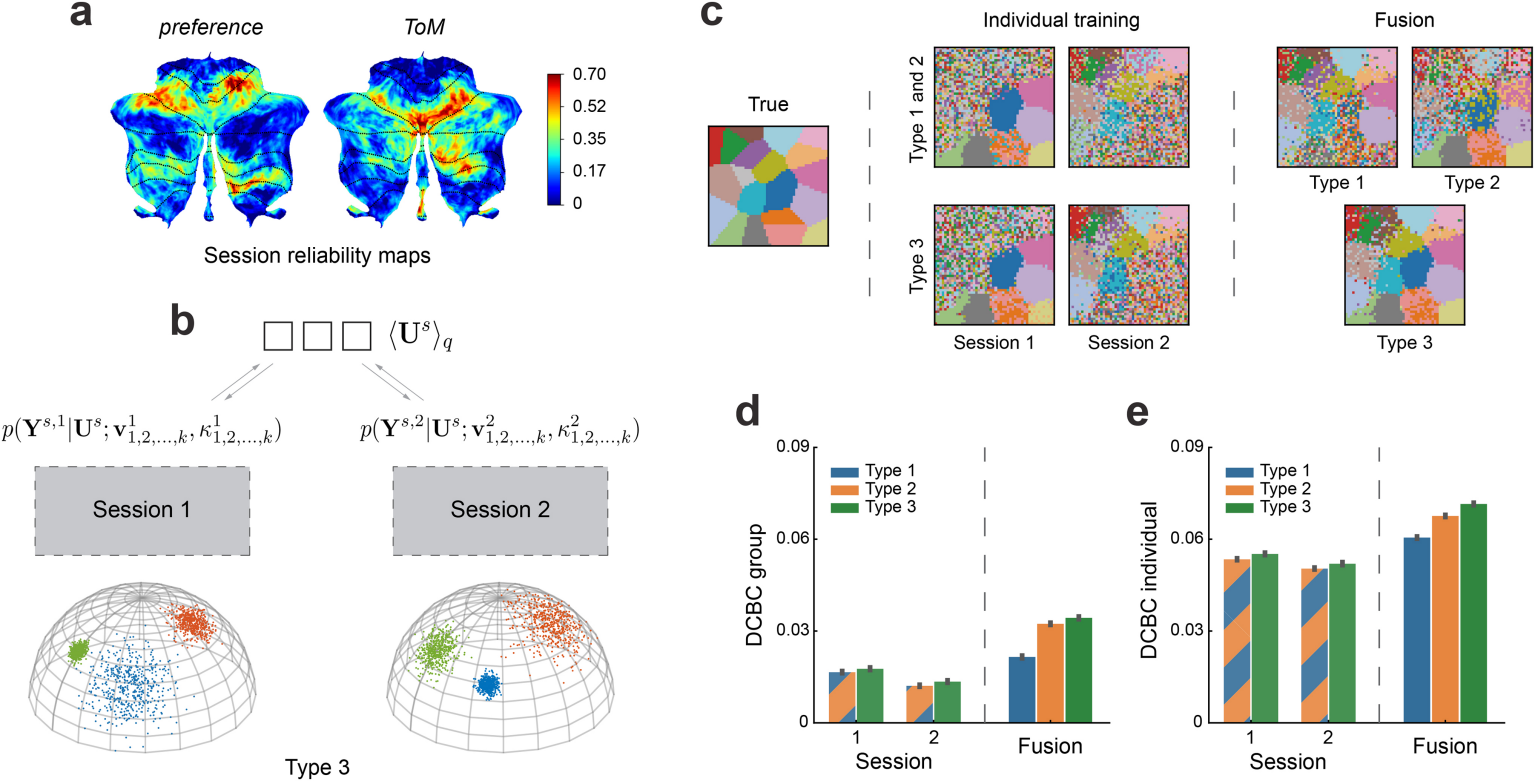
Simulation on two synthetic sessions fusion with different task activation. (a) Split-half reliability of two imaging sessions from the IBC dataset with different tasks (*preference*and*ToM*). (b) Type 3 model: different sessions are modeled using different emission models, and, furthermore, the concentration parameters$\kappa_{1,2,...k}$are estimated separately for each parcel. (c) The comparison of reconstruction performance when leaned on synthetic session 1 or 2 alone vs. learned by data fusion using Type 1, 2, or 3 models. (d) The mean DCBC value of the group map across sessions and model types. (e) The mean DCBC value of individual maps across sessions and model types. Error bars indicate SEM across 100 times simulation.

In empirically observed task-based fMRI data, however, the signal-to-noise level does differ not only between sessions or datasets, but also between different regions within the same session or dataset. Some sessions or datasets provide a better signal-to-noise ratio for some functional regions than others. For example (Fig. 4a), the “*preference*” session of the IBC dataset provided high within-subject reliability in the motor areas, whereas the “*theory-of-mind*” (*ToM*) session had high reliability in social-linguistic areas. Ideally, a probabilistic framework should account for these differences and optimally combine the region-specific strengths of each dataset. To this end, we introduced a third variant of our emission model (Type 3), which has a separate concentration parameter for each region and session (e.g.$\kappa_{1,2,...,k}^{1}$for session 1 and$\kappa_{1,2,...,k}^{2}$for session 2 inFig. 4b).

To test the ability of this model to pool information across distinct datasets with different types of information, we conducted a second simulation by randomly dividing functional regions into two groups. Instead of a common signal-to-noise level for all regions, we first created synthetic data in which one session had good signal-to-noise level in the first group and poorer signal-to-noise level in the other (Section 2.4). We reversed the assignment for the second synthetic session. When we trained the model on Session 1 or 2 alone, there was high uncertainty of the cluster assignment in the area with low signal-to-noise level (Fig. 4c—*Individual training*). This is expected, as the activation here was too weak to detect the boundaries reliably.

Importantly, when combining the two sessions, the functional boundaries that were not detected based on single sessions became visible (Fig. 4c—*Fusion*). However, both Type 1 and Type 2 models needed to compromise: when using session 1 to achieve parcellation of the lower right corner, the same weighting was applied to the upper left regions, decreasing the quality of the parcellation there. In contrast, model Type 3 allowed different concentration parameters in different parcels, using mostly information from session 1 for the lower right parcels and mostly information from session 2 for the upper left regions. The quantitative evaluation of DCBC (Fig. 4d,4e) suggests a clear advantage of model Type 3 over Type 2 model for both the group (improved 0.002, SD=$3.324\times 10^{-3}$) and individual parcellation (improved 0.004, SD=$3.831\times 10^{-3}$). This advantage is also shown when calculating the average reconstruction error relative to the true maps (SupplementaryFig. S4b). We also verified Type 3 model did not perform worse than Type 2 when two sessions had the same signal-to-noise level across all functional regions (see SupplementaryFig. S5). Overall, the model with region-specific concentration parameters showed clear advantages when aggregating across sessions that differ not only in their overall signal-to-noise level, but also in what regions they specifically provide information for.

### 3.4 Model performance on real data and the choice of atlas resolution K

Having established that our model works as expected for the fusion of synthetic datasets, we tested it on real imaging data. Here, we first used the IBC dataset. This dataset is ideal for testing the integration of data from different sessions across the same participants, as it consists of 14 sessions, some of which have similar tasks while others do not (Pinho et al., 2018,^2020^,^2024^). We tested the different model types, each time fusing two IBC sessions ($C_{14}^{2}=91$combinations) to learn a new probabilistic group map with 17 parcels. The learned group map was then evaluated on six other functional task-based fMRI datasets (see Tabel 1) in terms of their group and individual parcellations. For the latter, we split each dataset into two halves. The first half was used to infer the individual parcellations${\text{U}}^{s}$for the participants of that dataset. The other half was used as a test set to calculate the DCBC value (Section 2.5). We then reversed the role of the two halves and averaged performance across the two cross-validation folds.

**Fig. 5.**
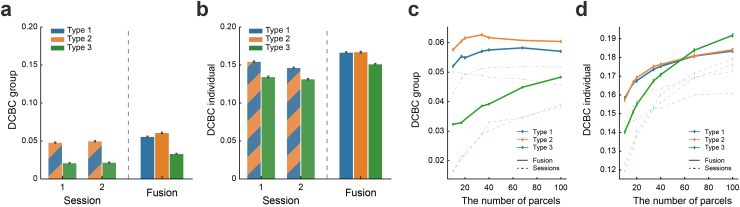
DCBC evaluation of a probabilistic group map (K = 17) learned on two IBC sessions alone compared with the fusion of the two sessions. (a) Mean DCBC value of the group map across the remaining six other datasets. Data are averaged across all 91 two-session combinations used for atlas training. (b) Mean DCBC value of individual parcellations of the six other datasets. (c) Mean DCBC value of the group map as a function of the number of parcels. (d) Mean DCBC value of the individual maps as a function of the number of parcels. Again, all results averaged across all 91 two-session combinations. All error bars indicate the SEM across the evaluation subjects across the six task-based fMRI datasets.

We first confirmed that the performance of the probabilistic group map learned by fusion across sessions outperformed the group maps learned from single sessions. Specifically, all fusion parcellations showed substantial improvement (Fig. 5a) over the better of the two single-session maps (for all types,$t_{98}>12.282,p<1.513\times$$10^{-21}$). This improvement also held for individual parcellations (Fig. 5b, for all types,$t_{98}>9.353,p<3.079\times 10^{-15}$). Additionally, we found the group parcellations learned using session-specific emission models (Type 2) showed significantly better performance than the ones learned by concatenating the data (Type 1) ($t_{98}\;=\;\;13.287,p=1.196\times$$10^{-23}$).

Against our expectations, however, model Type 3 performed substantially worse on real data when compared with model Type 2 for both group ($t_{98}\;=\;-16.765,p=1.521\times$$10^{-30}$) and individual ($t_{98}\;=\;\;-6.269,p=9.807\times 10^{-9}$) parcellations. This behavior differed markedly from our simulation results (Fig. 4), where model Type 3 performed consistently better. Further simulations suggested that this behavior can be explained by the choice of the number of parcels (K): whenKwas close to or higher than the true number of parcels, model Type 3 outperformed model Type 2. If, however,Kwas chosen to be smaller than the trueK, model Type 3 started to yield inferior performance (SupplementaryFig. S6). In such cases, one parcel in model Type 3 typically had a very low concentration parameter, effectively capturing all voxels that are unexplained by the model. Model Type 2 constrains all functional regions to have the same concentration parameter, preventing the model from developing a “residual” parcel.

This idea suggests that model Type 3 should improve or even outperform model Type 2 whenKincreases and approaches the true number of parcels. Unlike the simulation, the true number of parcels in real data is unknown. We, therefore, estimated the fusion models on every pair of two IBC sessions usingK=(10,17,20,34,40,68,100). The evaluation results (Fig. 5c, d) indicated that the performance of model Type 3 indeed improved with increasingK. This improvement was also clearly observed in individual parcellations (Fig. 5d), where the DCBC evaluation of model Type 3 became as good as model Type 2 aroundK=60and showed a significant advantage atK=100($t_{98}\;=4.115,p=8.059\times 10^{-5}$). A similar pattern exists in the group map evaluation where the averaged DCBC value of 100 parcels substantially improved compared with the ones with only 10 parcels ($t_{98}\;=\;\;28.191,$$p=8.215\times 10^{-49}$). For up to 100 parcels, the fusion parcellation from model Type 3 did not appear to be superior to the one from model Type 2 in group evaluation; however, we found this to be the case when considering more datasets (seeFig. 6e).

**Fig. 6.**
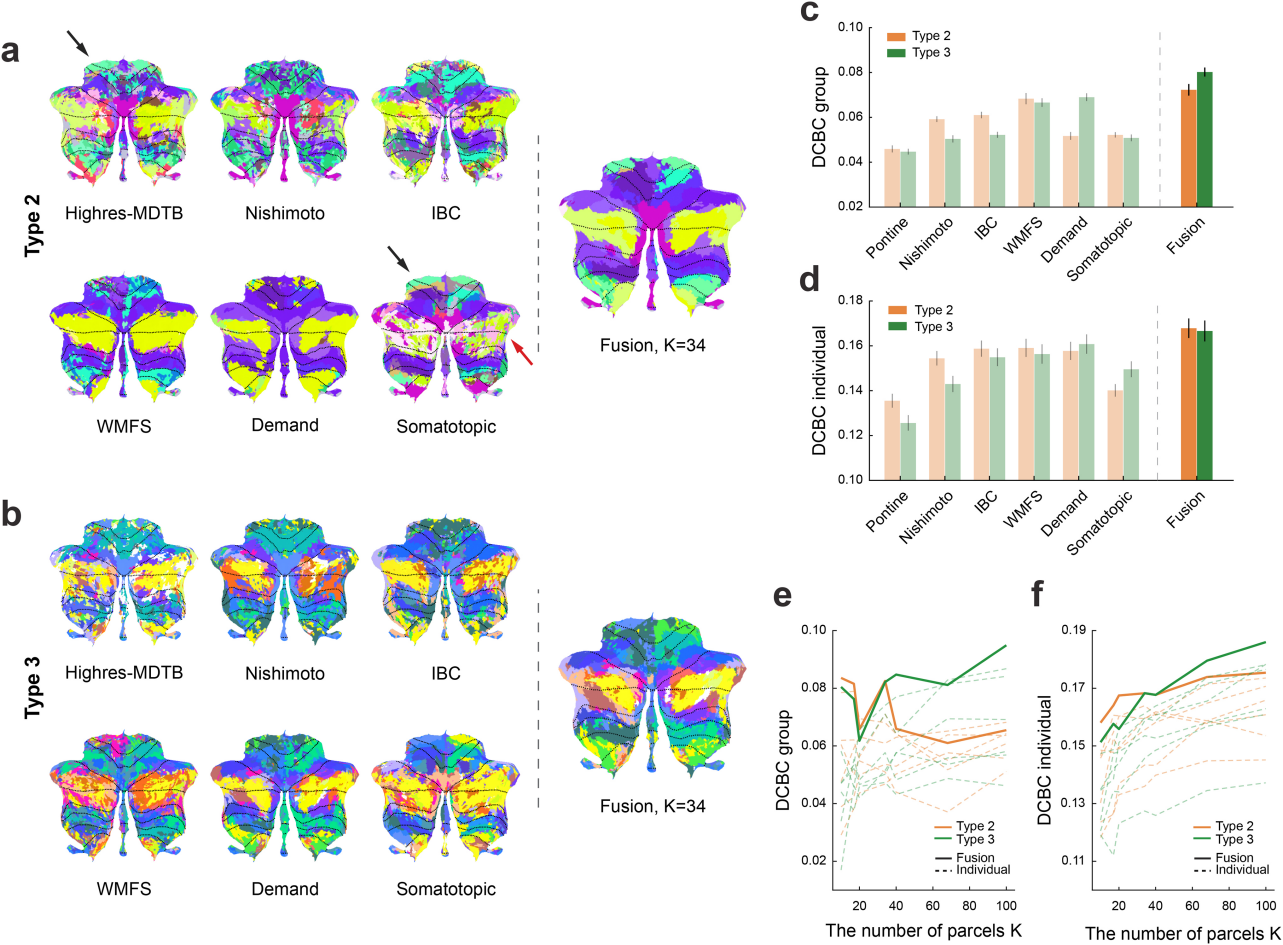
Comparison of cerebellar parcellations learned by Types 2 and 3 fusion models using 6 functional task-based datasets. (a) The group parcellation maps (K=34) derived from each individual dataset alone or through datasets fusion using Type 2 model. The black and red arrows point to the foot region and Crus I/II of the cerebellum, respectively. (b) Same as (a), but using Type 3 model. (c) Mean DCBC value of the group parcellation maps across subjects in the test dataset. Results are averaged acrossK=10to 100. (d) Mean DCBC value of the individual parcellation maps across subjects in the test dataset. (e) Mean DCBC value of the group map forK=10to 100. (f) Mean DCBC value of the individual map forK=10to 100.

Overall, across analysis scenarios, we confirm that estimating separate concentration parameters for each session (Type 2) leads to better data fusion on real fMRI data. Additionally allowing a region-specific concentration parameter (Type 3) has both advantages and disadvantages: If the model assumes a large number of parcels, parcellations can improve. If, however, the assumed number of parcels is low, performance appears to be better when constraining the concentration parameter to be the same across regions.

### 3.5 The fusion atlas shows combined strengths across different task-based fMRI datasets

Finally, we tested the framework for its main intended purpose: namely to fuse multiple different task-based datasets into a single parcellation. To test this ability, we trained our fusion model on six of the seven task-based fMRI datasets (Table 1), reserving the*MDTB*dataset for testing. The resultant group maps of both models Type 2 and 3 showed the combined strength of the maps trained on individual datasets. For example, only the group maps derived from the*Somatotopic*and*Highres-MDTB*datasets delineated the foot region of the cerebellum (Fig. 6a, black arrows), while the ones derived from other datasets did not. The Fusion map veridically retained this region. In contrast, the parcellation based on the*Somatotopic*dataset did not show a good parcellation of lobules Crus I and II (Fig. 6a, red arrow), but here the fusion map used information from other datasets.

To evaluate the parcellations quantitatively, we calculated the DCBC on the left-out*MDTB*dataset (Fig. 6c, d). For the individual parcellations, we split the MDTB dataset into an individual training and test set (see methods). Averaged across allK′s, all parcellations showed positive DCBC values, which means that the functional boundaries learned from any of the datasets generalized to some degree to the*MDTB*dataset. The best DCBC among parcellations trained on a single dataset was for the*WMFS*dataset for model Type 2 and for the*Demand*dataset for model Type 3. When we evaluated the fusion parcellations, we found considerable improvements for both models compared with the best individual parcellation: For the fused parcellation using model Type 2, both the group DCBC ($t_{23}\;=\;\;2.339,p=2.840\times 10^{-2}$) and the individual DCBC ($t_{23}\;=\;\;3.173,p=4.248\times 10^{-3}$) were considerably better than for*WMFS*. Similar improvement could be observed for model Type 3, where the fused parcellation significantly outperformed the best single-dataset parcellation (*Demand*) in terms of both the group ($t_{23}\;=\;\;7.049,p=3.503\times 10^{-7}$) and individual ($t_{23}\;=\;\;3.219,p=3.800\times 10^{-3}$) DCBC value.

Finally, we compared the fusion across the six task-based fMRI datasets directly between model Types 2 and 3. ForK=10, both group and individual DCBC (Fig. 6e, f) were higher for model Type 2 than for model Type 3 (group:$t_{23}\;=\;\;0.726,p=0.475$; individual:$t_{23}\;\;=1.842,$p=0.078). But whenKincreased to 100, the fusion parcellation for model Type 3 became substantially better than model Type 2 (group:$t_{23}\;=\;\;4.551,p=1.426\times 10^{-4}$; individual:$t_{23}\;=\;\;2.468,p=2.144\times 10^{-2}$). Similar results can also be obtained if evaluation is performed on the HCP resting-state data (SupplementaryFig. S7).

### 3.6 Integrating resting-state data into the task-based parcellation

Lastly, we tested the ability of our framework to fuse resting-state and task-based data into a single parcellation atlas. To do so, we used the cortical connectivity profile for each cerebellar voxel derived for 50 participants from the HCP*Unrelated 100*dataset (seeSection 2.2). As we wanted to evaluate performance on a large range of task-based datasets, we used each of the seven task datasets for testing and excluded that dataset from the model training.

**Fig. 7.**
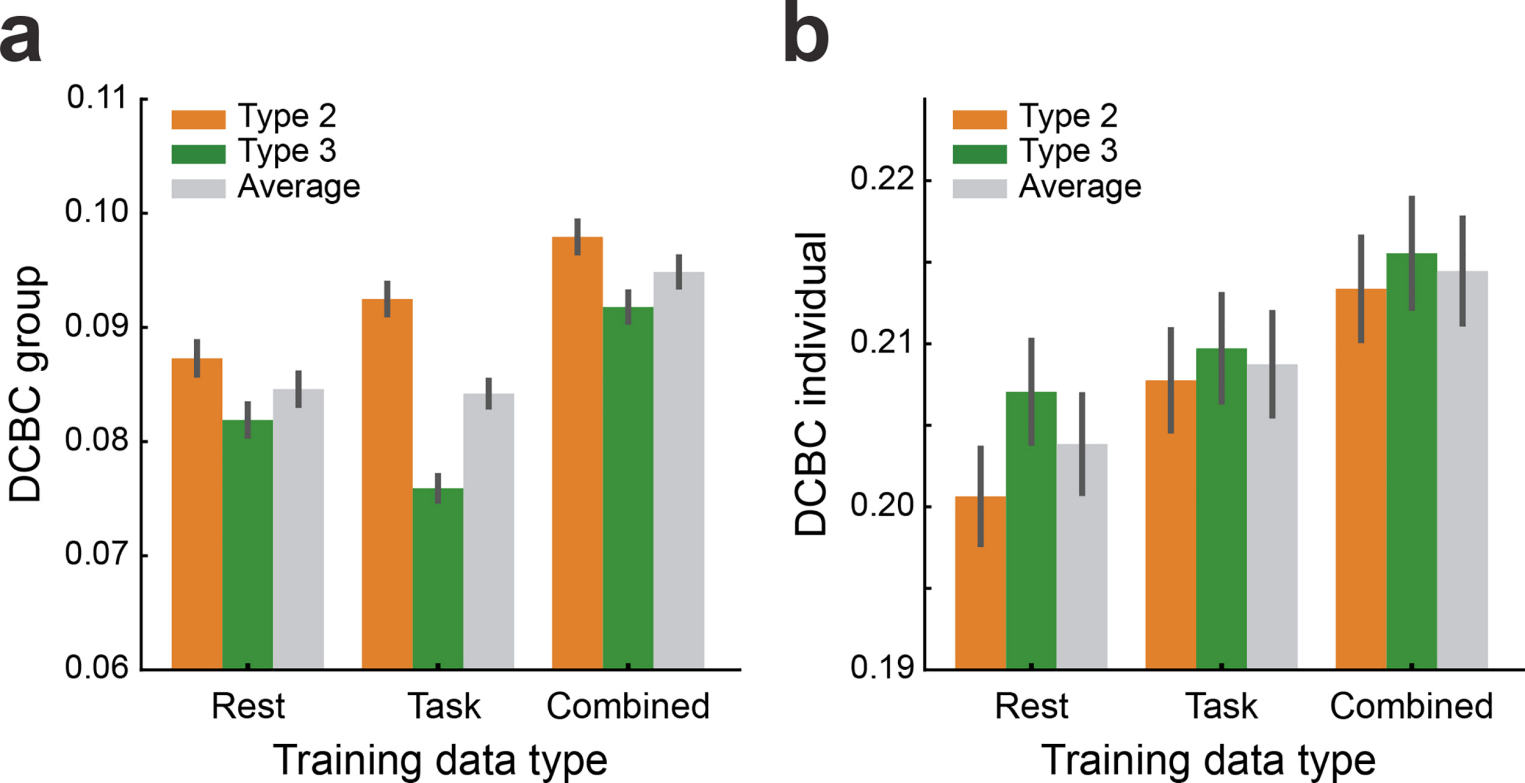
**Performance of cerebellar group parcellations derived from resting-state data only, task-based data only, or the combination of both.**Probabilistic parcellations were learned using Type 2 (orange) or 3 (green) models. The gray bar indicates the averaged performance across the two models.**(a)**Mean group DCBC, and**(b)**mean individual DCBC evaluated on the task-based datasets in a leave-one-dataset out fashion. Error bar indicates the SEM across all 111 subjects of the 7 task datasets. Results are averaged across all tested levels ofK=10to 100.

Averaging the DCBC evaluations across models (Type 2 and 3) and allK′s, the models trained on the combination of resting-state and task-based datasets outperformed the ones trained on resting-state or task-based datasets alone. For the group parcellation (Fig. 7a), the combined model was significantly better than the one trained on the resting-state ($t_{110}\;=\;\;\;6.349,p=4.983\times 10^{-9}$), and six task-based datasets ($t_{110}\;=\;\;3.886,p=1.745\times 10^{-4}$). Similar results were found for individual parcellations (Fig. 7b, vs. resting-state alone:$t_{110}\;=\;\;7.625,p=9.287\times 10^{-12}$, vs. task-based alone$t_{110}\;=\;\;7.254,p=6.027\times 10^{-11}$).

## 4 Discussion

We developed a hierarchical Bayesian framework that solves two important problems in brain parcellation: First, by using dataset-specific emission models, the framework can optimally integrate information across many, quite heterogeneous, datasets. Here we showed an example of the integration of a diverse set of task-based fMRI datasets and resting-state data. Second, because the framework directly models individual differences in brain organization, it provides not only a probabilistic group atlas, but also allows the user to obtain an optimal estimate of brain organization for new individuals.

### 4.1 Learning functional brain parcellations across datasets

While most of the current brain parcellations are generated using functional resting-state fMRI data, a number of studies (Cole et al., 2014;King et al., 2019) suggest that boundaries derived using resting-state data can differ systematically from those measured during task performance. One possible interpretation of this finding is that the boundaries of functional regions truly shift depending on the task the person performs (Salehi et al., 2020). However, our results also show clearly that models trained on specific task-based datasets are able to predict functional boundaries in other task-based datasets substantially above chance (Fig. 6c, d). This clearly argues that there is a basic common organization that is task invariant (King et al., 2019;Tavor et al., 2016). Following this viewpoint, different task-based or resting-state datasets highlight different aspects of these stable boundaries. This is obviously true for two task sets that emphasize different aspects of mental function (seeFig. 4a), but also applies to resting-state data. For example, in resting-state data, left- and right-hand regions are usually highly correlated and often end up in the same parcel. However, when using a task set that contains both left and right unimanual movements, the two regions are readily dissociated (King et al., 2019). Therefore, the integration of data from a large array of tasks promises a more representative map of brain organization.

Because there is no single task-based dataset that covers all mental functions in a large number of participants, our main goal with this paper was to develop and validate a framework that allows us to fuse data from a growing number of deep-phenotyping task-based datasets (Assem et al., 2024;King et al., 2019;Nakai & Nishimoto, 2020;Pinho et al., 2018,^2020^). Even though our framework shares substantial similarities with a previous hierarchical Bayesian model for brain parcellations (Kong et al., 2018), this model was targeted at resting-state data only, but was not able to also integrate different types of task-based datasets. Here we solve this problem by deploying a series of emission models, each one learning the expected response for each brain region and their variability. The integration across datasets is achieved through a common spatial arrangement model, which characterizes the variability of the functional organization across individuals. As shown in the simulations (Sections 3.2and3.3), this allows us to integrate the strength of different datasets without inheriting their weaknesses. We can now deploy this framework to an increasing number of datasets, including “wide” datasets with many participants (King et al., 2019), and “deep” datasets with only a few participants but a detailed characterization of each studied individual (Nakai & Nishimoto, 2020;Pinho et al., 2018,^2020^). We provide a practical example of how to use the framework to learn a new probabilistic atlas across various datasets at hierarchbayesparcel.readthedocs.io.

### 4.2 Individual vs. group parcellation maps

Group parcellation maps identify patterns of functional organizations that are common and consistent across individuals. Group parcellations are in common use, as they provide a consistent framework to analyze and report functional imaging data, and can be applied using only the anatomical image from the individual. However, the boundaries between functional regions vary substantially across individual brains (Braga & Buckner, 2017;Gordon et al., 2017;Kong et al., 2021), possibly biasing subsequent analysis (Bijsterbosch et al., 2018,^2019^). Therefore, using individual brain parcellations has the potential to improve the precision and quality of subsequent analyses. A major limitation, however, is that a substantial amount of individual data is necessary to derive an individualized map of sufficient quality (Marek et al., 2018). In our study, we found that 60 min of individual data were required to reach the same performance as the group map, and more than 110 min of data were necessary to substantially outperform it (seeSection 3.1). For most studies, acquiring this amount of data for an individual functional localizer would be prohibitive, explaining the persistent popularity of group maps.

Different from previous approaches to derive individual parcellations (Salehi et al., 2018;Thirion et al., 2024;Zhang et al., 2021), our approach performs a principled (Bayesian) integration between a group atlas and the evidence from the individual functional localizer scan, weighting each according to the respective uncertainty, see alsoKong et al. (2021). Even when using a very short functional localizer (10 min), the resultant individual parcellation outperforms the group map. An example of how to use the framework to derive individual parcellations for a new set of participants using an existing atlas can be found at hierarchbayesparcel.readthedocs.io.

### 4.3 Comparing dataset-specific and regions-specific concentration parameters

The concentration parameter (κ) in each emission model dictates how strongly the respective dataset is weighted, both when learning to determine the group parcellation map and when integrating individual data with the existing group map for individual parcellation. In this paper, we tested three ways of estimating this concentration parameter: (a) we simply concatenated all sessions for each subject, giving the entire dataset a single concentration parameter (Type 1); (b) we used a separate emission model and, therefore, a separate concentration parameter for each session (Type 2); and (c) we used a separate concentration parameter for each session and region (Type 3).

We first showed that model Type 2 performed better than model Type 1 in capturing different levels of measurement noise from different sessions in both simulation and real data (Sections 3.2and3.4). However, when we compared Type 2 (dataset-specific) and Type 3 (region-specific) models, we found that each had specific advantages, which were also dependent on the choice of the number of parcelsK(Section 3.4). When the number ofK′swas large, the region-specific model led to better parcellations, it could account for the fact that some sessions contain tasks that provided signals in some areas, while other sessions highlighted other areas, a behavior clearly visible in the IBC dataset (Fig. 4a). However, when the assumed number of parcels (K) was smaller, one region would be estimated to have a very low concentration parameter, such that it could model all the residual, nonexplained regions. Such a residual region led to a more fragmented group parcellation (Fig. 6b) and an impaired evaluation of the independent data. Constraining the concentration parameters to be the same across all regions (model Type 2) prevented this from happening. The choice of emission model (Type 2 or Type 3), therefore, will depend on the desired granularity of the parcellation and likely also on the amount and quality of the available data. Our framework offers both implementations, allowing the user to choose the correct algorithm in a context-specific manner.

### 4.4 Choice of the number of parcels

When creating a new parcellation atlas, the user needs to decide on the number of parcels,K. This is a notoriously difficult question, which, for the human brain, likely does not have a single correct answer. In our paper, we started with 10 and 17 parcels, as these are used in previous studies (Buckner et al., 2011;King et al., 2019), and then doubled these numbers twice. While the individual parcellations still seem to improve atK=100since weaker individual-specific functional boundaries are detected with finer granularity, the DCBC starts to decline and stabilizes forK>200(not shown).

This does not mean, however, that parcellations with a higher number of parcels are always preferable, even if they perform better. Depending on the purpose of the study, the type of data that is being analyzed, and the amount of individual data, a lower number of parcels may provide a more succinct and understandable summary of the data. Our current paper focuses on validating the computational framework—therefore, we attempted to show results across a range of granularities. In our companion paper (Nettekoven et al., 2024), we present a single new multiresolution atlas for the cerebellum that starts withK=68as the finest resolution, and then uses a hierarchically nested scheme to combine these parcels into larger regions. We believe that this scheme does provide a good balance between detailed prediction performance and simplicity.

### 4.5 The choice of training datasets

In principle, our proposed framework can integrate any type of data, including different types of structural, genetic, or functional data. Depending on the data type, new emission models may have to be created (the repository provides a von Mises–Fisher mixture model, a Gaussian mixture model, and a multinomial model for discrete labels, seehttps://hierarchbayesparcel.readthedocs.io). However, care needs to be taken when combining different data modalities, as each may reveal different types of brain organization.

In our paper, we combined different task-based and resting-state fMRI datasets. We show that each modality can predict functional boundaries in the other modality well above chance (Fig. 7and SupplementaryFig. S8). However, visual inspection of the two parcellations (see SupplementaryFig. S9a, b) also reveals some systematic differences (Cole et al., 2014;King et al., 2019). Our Bayesian framework simply weights each dataset according to its reliability, ignoring any differences in the mean organization. Because different datasets will emphasize slightly different sets of functional boundaries, each type of dataset will bias the final parcellation in a specific direction (Nettekoven et al., 2024). Consequently, a single large dataset could dominate the group map, possibly reducing the predictive performance for other datasets. It is, therefore, important to achieve a good balance between resting-state datasets, which can be very large, and different task-based datasets, each potentially highlighting a specific cognitive domain (Salehi et al., 2020). Where this balance lies, or whether it is preferable to have different brain parcellations for different functional states, remains an open research question that is outside the scope of this paper.

### 4.6 Limitations and further developments

The main purpose of this paper is to introduce and validate a hierarchical Bayesian framework that optimally fuses information from different types of fMRI datasets. In a companion paper (Nettekoven et al., 2024), we use this framework to develop a new parcellation of the cerebellum that has a nested structure over three levels of granularity and matched regions across the left and right hemispheres. We also provided a careful characterization of the new regions and extensively compared the resultant atlas against existing nonprobabilistic parcellations of the cerebellum, using both DCBC and other evaluation criteria.

In this work, we focus mostly on the use of our framework to learn a new probabilistic group atlas. An important practical application, however, is to derive individual parcellations for new subjects using a new dataset, using an*existing*and established probabilistic atlas. For this purpose, the new dataset would need to serve as an individual training (or functional–localizer dataset). After the estimation of a new emission model for this dataset, the resultant individual parcellations can then be interpreted in the framework of the established atlas. This approach makes individual functional localization in new studies feasible, while still leveraging the insight won from all the other datasets. This has important applications for basic science, clinical studies, and ultimately individualized prognosis in medicine.

To optimize this approach, however, a number of open questions remain: Is it better to use resting-state data or a specific task-based dataset as functional localizer? If the latter, which combination of tasks would be optimal? What type of statistical model is optimal to obtain the most precise individual predictions? These questions could not be addressed within the scope of this paper, and await future investigation.

Finally, we have only used the independent spatial arrangement model in this paper, which in essence learns a probabilistic group atlas. Being able to leverage an increasing number of datasets, however, will hopefully allow further development of models that can learn spatial regularities in the arrangement of functional regions in the human brain. In our framework, we can also use models that make assumptions about the intrinsic smoothness of individual functional parcellations, such as a Markov Random Field (MRF) spatial prior (Kong et al., 2018;Ryali et al., 2013;Schaefer et al., 2018) with coupling parameters. As a further extension, deep generative models, such as a deep Boltzmann machine (Salakhutdinov & Hinton, 2009), provide a promising avenue to learn the complex short- and long-range dependencies in individual functional brain organization. We have already developed and tested such a deep Boltzmann machine as a spatial arrangement model in our framework (Chapter 4,Zhi, 2023). However, for the cerebellum, there was no benefit for modeling spatial dependencies between voxels. For cortical data collected at high resolution, we found a very slight (but not significant) advantage in modeling the spatial dependencies between brain locations. Therefore, we focus in this paper only on the independent spatial arrangement model. However, developing a spatially informed model is a promising avenue for further work, and our framework can easily be extended to incorporate such models.

## 5 Conclusion

This paper introduces and validates a hierarchical Bayesian parcellation framework for data fusion. Advancing on previous models (Kong et al., 2021), our framework can integrate different types of task-based datasets with resting-state data. Here, we have validated the framework using data from the human cerebellum—however, the same process can be repeated for any other brain structure.

We anticipate that this framework will be useful for two reasons. First, the model can provide individual functional parcellations for new subjects using very limited individual data. While normally individual parcellations require an extensive amount of data (Marek et al., 2018), our framework makes it feasible to derive an individual region definition of considerably better quality than a group map with 10 min of functional localizer data. Second, the framework allows the optimal fusion of functional insights using a range of different task-based datasets, thereby overcoming the limitation that current task-based datasets are restricted in terms of both the breadth of their task battery and the number of subjects. The framework accurately quantifies the different signal-to-noise levels across sessions and datasets, thereby providing an optimal weighting for each. The resultant maps possess a combined strength in detecting the detailed functional boundaries, outperforming the parcellations trained by single datasets.

## Supplementary Material

Supplementary Material

## Data Availability

The raw data for the fMRI studies used in this project are publicly available at the links listed inTable 1. The code for the hierarchical Bayesian parcellation framework is publicly available as the GitHub repositoryhttps://github.com/DiedrichsenLab/HierarchBayesParcel. The organization, file system, and code for managing the diverse set of datasets are available in a separate repositoryhttps://github.com/DiedrichsenLab/Functional_Fusion. The paper-specific code for generating the functional probabilistic parcellations for the cerebellum, as well as running the simulation presented in this paper, is available athttps://github.com/DiedrichsenLab/FusionModel.
