# Supplementary material for: A hierarchical Bayesian brain parcellation framework for fusion of functional imaging datasets
Source: Imaging Neurosci (Camb). 2025 Jan 2;3:imag_a_00408. doi: 10.1162/imag_a_00408 (PMC12319954; doi:10.1162/imag_a_00408)
Supplement: Supplementary Material [file imag_a_00408-supp.pdf]

Supplementary Materials and Figures

1. Parameter estimation of full model

In this section, we provide details of model parameter estimation for the full EM algorithm. The complete expected log-likelihood $\sum_s \langle \log p(\mathbf{Y}^s, \mathbf{U}^s; \boldsymbol{\theta}) \rangle_q$ can be decomposed into expected emission log-likelihood $\mathcal{L}_E$ and expected arrangement log-likelihood $\mathcal{L}_A$, where $\langle \cdot \rangle_q$ denotes the expectation with respect to distribution q. Similarly, the model parameter θ can be subdivided into θ_E and θ_A and can be estimated within their corresponding models (Methods 2.1). This unique model structure yields the following learning EM process:

Emission model E step. Suppose for a single dataset $\mathbf{Y}^n$ is a $S \times N \times P$ tensor for S subjects (S is the number of subjects in $\mathbb{S}$) of N data observations across P voxels. The brain activation of a voxel for a single subject $\mathbf{y}_i^s$ is a N-long vector. If the task design has repeated measurements of the same M conditions (e.g. in a single imaging run), the user can specify this over a $N \times M$ design matrix X (M is the number of unique task conditions). To account for the situation that $\mathbf{y}_i^s$ consists of multiple partitions, which could be imaging sessions or runs, we used an N-dimensional partition vector to divide N observation into J independent partitions. Therefore, if we combine the data across repeated measurements and different partitions, the resultant data $\tilde{\mathbf{y}}_i^s$ would be a sum of normalized data in each partition j as,

$$\tilde{\mathbf{y}}_i^s = \sum_j^J \|(\mathbf{X}_j^\top \mathbf{X}_j)^{-1} \mathbf{X}_j^\top \mathbf{y}_{i,j}^s\| \tag{S1}$$

However, we can also treat the different repetitions as independent observations, meaning that the resultant data is normalized to length 1 across J independent partitions. This is also the case with the Type 1 model, in which the imaging sessions are simply concatenated. Hence, the expected emission likelihood $\mathcal{L}_E$ of a mixture of k-classes vMF distribution in eq. 11 is modified and updated at $(t+1)$ iteration by:

$$\mathcal{L}_E{}^{(t+1)} = SPJ \sum_k \log c_M(\kappa_k^{(t)}) + \sum_{s \in \mathbb{S}} \sum_i^P \sum_k^K \langle \mathbf{u}_i^s(k) \rangle_q^{(t)} \kappa_k^{(t)} \mathbf{v}_k^{(t)\top} \tilde{\mathbf{y}}_i^s \tag{S2}$$

As a sufficient statistic, it should be noticed that the resultant summed vectors $\tilde{\mathbf{y}}_i^s$ become a M-dimensional vector but its magnitude is not 1 anymore. Therefore, the normalizing constant will be computed in M-dimensional correspondingly, denoted as $\log c_M(\kappa_k)$.

Arrangement model E step. Expanding eq. 10 and 7, the expected posterior under the proposal distribution q at $(t+1)$ iteration are updated as,

$$\langle \mathbf{u}_i^s(k)\rangle_q^{(t+1)} = p(\mathbf{u}_i^s = k|\mathbf{y}_i^s; \theta_A^{(t)}, \theta_E^{(t)}) \tag{S3}$$

$$= \frac{\exp(\langle \log p(\mathbf{y}_i^s|\mathbf{u}_i^s = k; \theta_E^{(t)})\rangle_q + \eta_i^{k(t)})}{\sum_j \exp(\langle \log p(\mathbf{y}_i^s|\mathbf{u}_i^s = j; \theta_E^{(t)})\rangle_q + \eta_i^{j(t)})} \tag{S4}$$

where η_i^k is defined in Methods.

Arrangement model M step. Expanding the expected arrangement log-likelihood in eq. 10, we obtain the derivatives with respect to the parameters $\theta_A := \{\eta_{i,k}\}$:

$$\frac{\partial \mathcal{L}_A}{\partial \eta_{i,k}} = \frac{\partial \sum_s \sum_i \langle \mathbf{u}_i^s(k)\rangle_q \cdot \eta_{i,k}}{\partial \eta_{i,k}} \tag{S5}$$

By setting this derivative to zero, we can obtain the following parameter updates:

$$\eta_{i,k}{}^{(t+1)} = \log \sum_s \langle \mathbf{u}_i^{s(k)}\rangle_q^{(t)} \tag{S6}$$

Emission model M step. To update the parameters θ_E of the vMF mixture in the M-step, we need to maximize $\mathcal{L}_E$ in respect to the parameters in vMF mixture $\theta_k = \{\mathbf{v}_k, \kappa_k\}$. First, we update the mean direction $\mathbf{v}_k$, where we get the intuitive update :

$$\mathbf{v}_k^{(t+1)} = \frac{\tilde{\mathbf{v}}_k}{r_k}, \qquad \text{where } \tilde{\mathbf{v}}_k = \sum_s \sum_i \langle \mathbf{u}_i^{s(k)}\rangle_q^{(t)} \cdot \tilde{\mathbf{y}}_i^s; \quad r_k = ||\tilde{\mathbf{v}}_k|| \tag{S7}$$

The updates of the concentration parameters κ_k are more difficult in particular for high dimensional problems, since it involves the inverting ratio of two Bessel functions. Therefore, we here use an approximate solution suggested by Banerjee et al. (2005) and Hornik and Grün (2014). In our specific case, we want to integrate the evidence across $s = 1, ..., S$ subjects and $i = 1, ..., P$ voxels, with each subject and voxel may have J_i^s partitions. Under this assumption, we can **(1)** learn a common concentration parameter κ across classes by restricting κ_k to be equal, as:

$$\kappa^{(t+1)} \approx \frac{\bar{r}M - \bar{r}^3}{1 - \bar{r}^2} \tag{S8}$$

$$\bar{r} = \frac{\sum_k^K \| \sum_s^S \sum_i^P \langle \mathbf{u}_i^{s(k)} \rangle_q^{(t)} \cdot \tilde{\mathbf{y}}_i^s \|}{\sum_s^S \sum_i^P J_i^s} \tag{S9}$$

which is used in Type 1 and Type 2 model learning.

Alternatively, we can **(2)** learn k-class specific κ_k by relaxing the constraint as:

$$\kappa_k^{(t+1)} \approx \frac{\bar{r}_k M - \bar{r}_k^3}{1 - \bar{r}_k^2} \tag{S10}$$

$$\bar{r}_k = \frac{\| \sum_s^S \sum_i^P \langle \mathbf{u}_i^{s(k)} \rangle_q^{(t)} \cdot \tilde{\mathbf{y}}_i^s \|}{\sum_s^S \sum_i^P \langle \mathbf{u}_i^{s(k)} \rangle_q^{(t)} \cdot J_i^s} \tag{S11}$$

which will be used as the parameter estimates for the Type 3 regions-specific emission model.

2. Generation of simulated parcellations

For the simulation in Results 3.2 and 3.3, we produced artificial individual brain parcellation maps from a Potts model (Wu, 1982) defined on a 50×50 grid. Each grid point represented a brain location and could take one of K values. We first generated an artificial smooth group probability map (Fig. S3a) by selecting K centroids μ_k at random locations, and assigning the bias parameters of the spatial arrangement model $\eta_{i,k}$ for the node a location x_i to be:

$$\eta_{i,k} = -\frac{|x_i - \mu_k|_2^2}{2\sigma_\mu^2} \tag{S12}$$

where σ_μ^2 controls the smoothness of the group map (see Supplementary Fig. S3b).

The individual maps $\mathbf{U}^s$ were then sampled from the Potts model where the local probability $p(\mathbf{u}_i)$ at each node dependent on the vector of bias parameters as well as the current state of the neighboring nodes (N).

$$p(u_{i,k}) = \text{softmax}(\eta_{i,k} + \sum_{j \in N} \psi_{i,j,k}), \tag{S13}$$

$$\text{where } \psi_{i,j,k} = \exp(\theta_w \cdot \mathbf{u}_{i,k} \mathbf{u}_{j,k} \cdot w_{i,j}) \tag{S14}$$

The pairwise weight of two vertices $w_{i,j}$ ($w_{i,j} = w_{j,i}$) indicates whether i and j are neighbouring vertices ($w_{i,j} = 1$ if i and j are neighbours; $w_{i,j} = 0$ otherwise). The temperature parameter θ_w controls how strong the spatial co-dependence between neigh-

bouring vertices is. A higher θ_w encourages that the two neighbouring nodes are more likely to be assigned to the same parcel, enforcing the overall local smoothness of the map (Supplementary Fig. S3c). Ultimately, the individual maps were generated using vertex-wise Gibbs sampling after a burn-in of 20 iterations across all vertices.

For the simulation in Results 3.2 and 3.3, the bias terms for the Potts model were generated with $\sigma_\mu^2 = 120$. Then, we sampled 10 individual maps $\mathbf{U}^s$ from the group map with local connection weights $w_{i,j} = 1.5$.

3. Expected reconstruction error

For the ground-truth simulations, another evaluation metric is to calculate the absolute reconstruction error between the true parcellation $\mathbf{U}$ and the estimated parcellations $\hat{\mathbf{U}}$ which inferred from the training data. Traditionally, the absolute reconstruction error is calculated between the hard parcellations. But in our project, we can also use a probabilistic version of this evaluation to calculate the mean expected reconstruction error under the probability distribution of the probabilistic parcellation across all brain locations, which is defined as:

$$\langle \bar{\epsilon}_{|\mathbf{U}-\hat{\mathbf{U}}|} \rangle_q = \frac{1}{PS} \sum_s^S \sum_i^P |\mathbf{u}_i^s - \langle \mathbf{u}_i^s \rangle_q| \tag{S15}$$

where the $\mathbf{u}_i^s$ represents the true parcel assignment i in subject s , as a one-hot encoded vector. Correspondingly, $\langle \mathbf{u}_i^s \rangle_q$ is the expected parcel assignment under the expectation q.

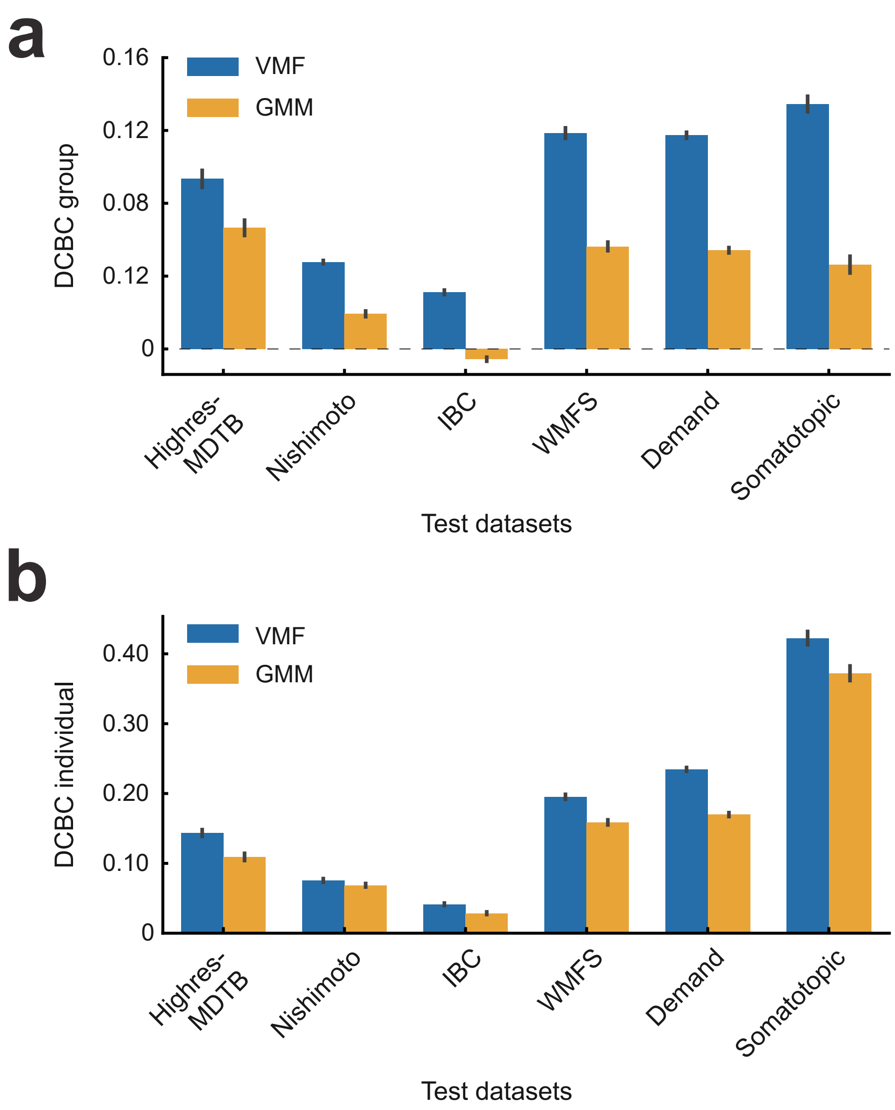

Figure S1: **Comparison of the performance between the parcellations derived from Gaussian Mixture Model (GMM) and von Mises-Fisher Mixture model (VMF). (a)** The averaged DCBC value of the group parcellation maps trained by GMM or VMF mixture model across subjects in the test dataset. **(b)** The averaged DCBC value of the individual parcellation maps trained by GMM or VMF mixture model across subjects in the test dataset.

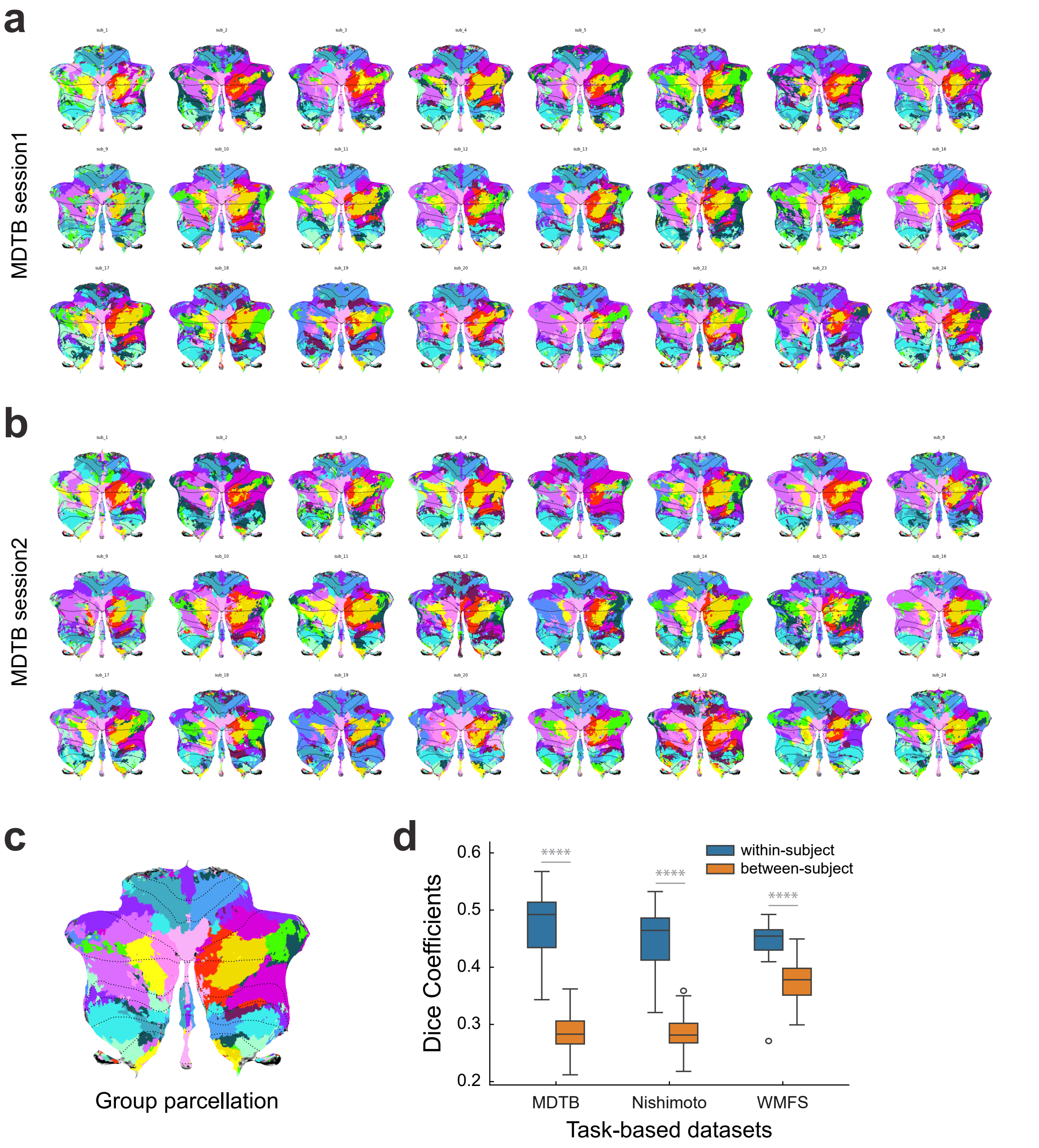

Figure S2: **The reproducibility test of the individual parcellations.** The individual parcellation (K=17) derived from MDTB task datasets **(a)** session 1 (task set A). **(b)** session 2 (task set B). Network colors are matched across subjects and aligned to the group map. **(c)** the group parcellation (K=17) derived from MDTB task datasets **(d)** the box plot of Dice coefficients between the individual parcellations of within-subject vs. between-subject derived from different task-based datasets, where 0 indicates total misalignment, 1 means perfect match.

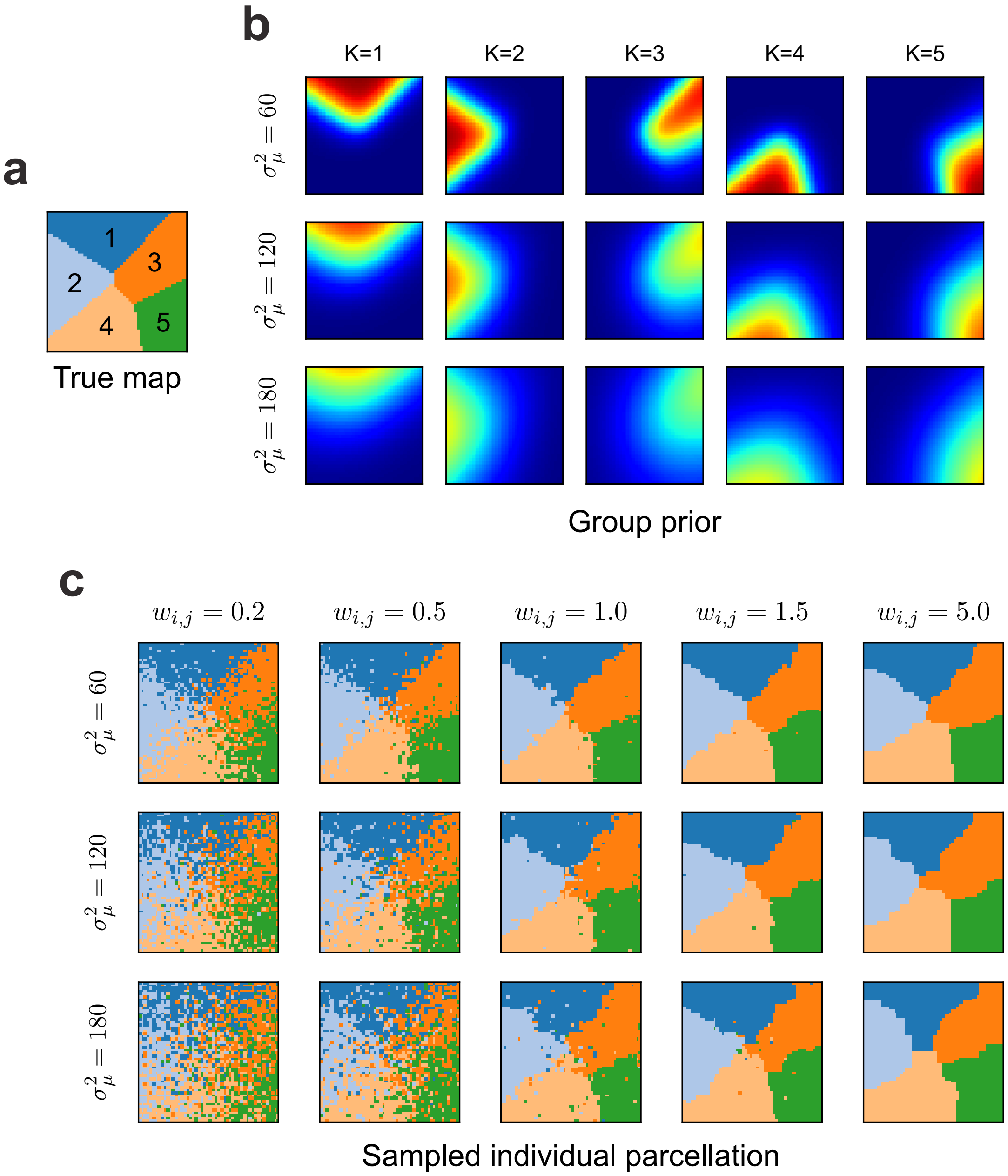

Figure S3: **The synthetic dataset.** **(a)** The random true group map with 5 parcels. **(b)** The group prior controlled by smoothing kernel at different levels for all 5 classes. **(c)** The example individual parcellation maps generated by different parameters

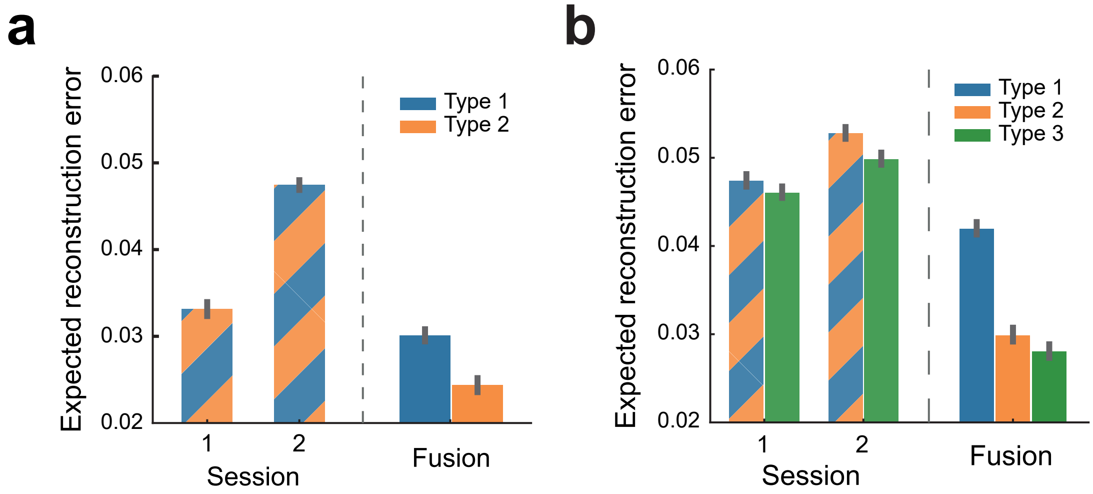

Figure S4: **The reconstruction error of the simulations in Results 3.2 and 3.3.** **(a)** the mean expected reconstruction error of the individual parcellations (Supplementary Materials 3) trained on synthetic session 1 or 2 alone vs. the ones trained by datasets fusion using type 1 or 2 models. **(b)** the mean expected reconstruction error of the individual parcellations trained on synthetic session 1 or 2 alone vs. the ones trained by datasets fusion using type 1, 2 or 3 models.

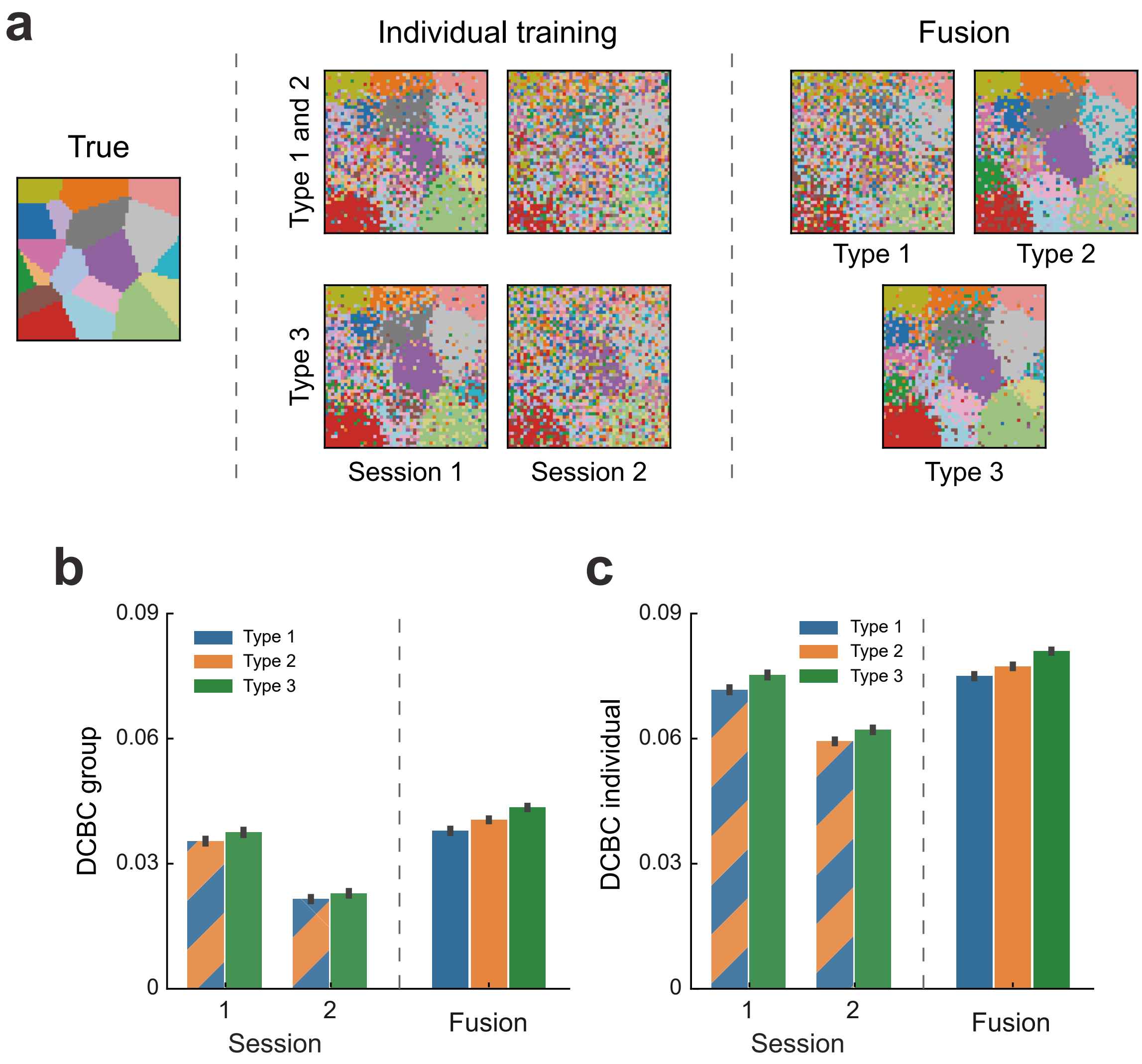

Figure S5: **Simulation on two synthetic sessions fusion with similar task activation using Type 1, 2, and 3 emission models.** **(a)** The comparison of model reconstruction performance of group parcellations learned on synthetic session 1 or 2 standalone vs. the ones learned fusion using type 1, 2, or 3 models. **(b)** The mean DCBC value of the group map learned from session 1 or 2 only or learned by fusion using type 1, 2, or 3 models. **(c)** The mean DCBC value of individual maps across all participants when learned from session 1 or 2 only or learned by fusion using type 1, 2, or 3 models. Error bars indicate SEM across 100 times simulation.

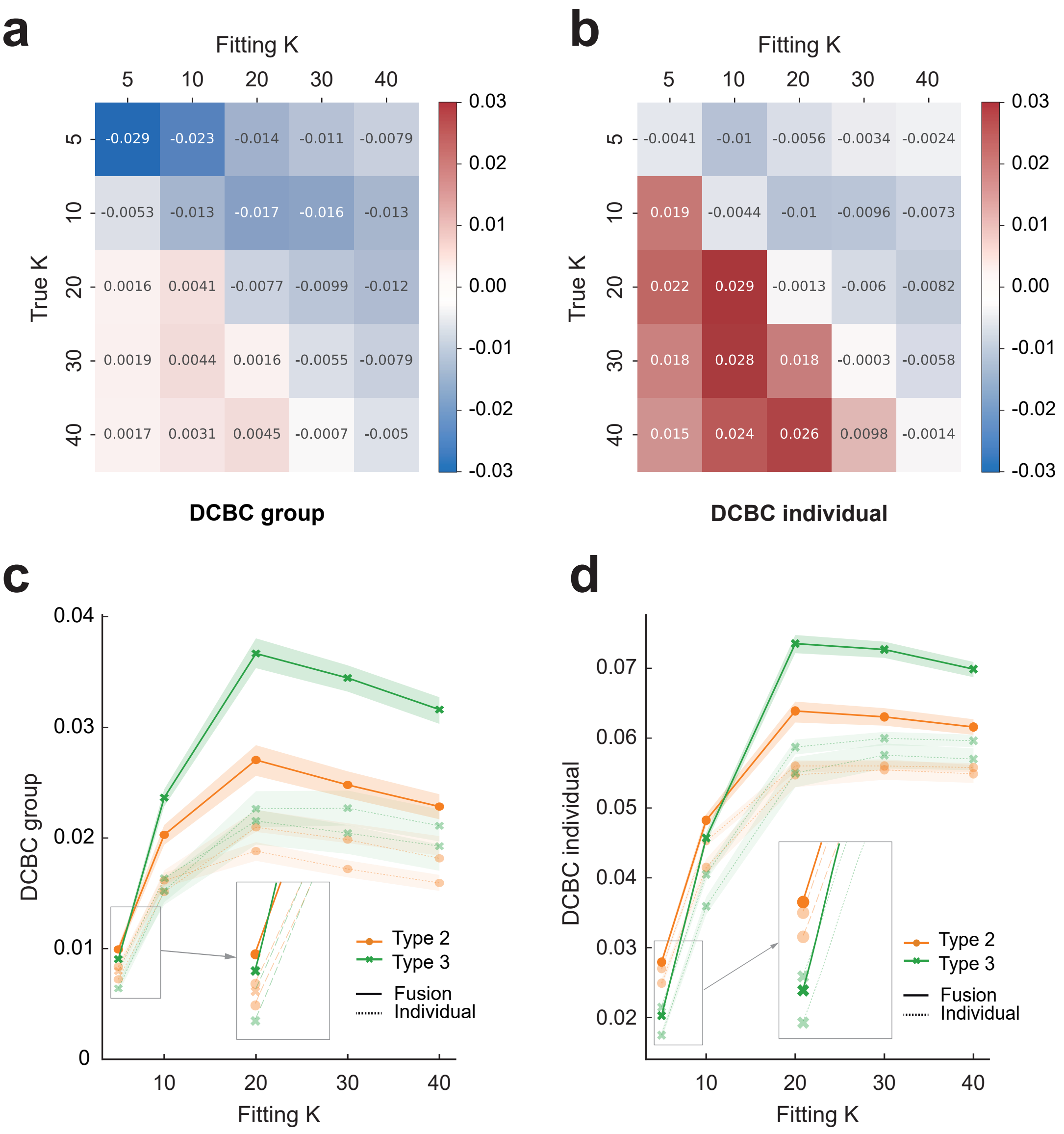

Figure S6: **Comparing the performance of Type 2 and 3 models when the number of parcels K used for fitting is different from the true K in the simulation.** **(a)** The difference of the mean DCBC value between the group map trained on a synthetic dataset using Type 2 and Type 3 models with different fitting K and ground true K, which tested on an independent synthetic test set. A positive value on the grid indicates the Type 2 model outperforms the Type 3 model, while negative values mean the opposite. **(b)** The difference of the mean DCBC value between the individual maps. **(c)** The mean DCBC value for the group map learned from individual synthetic datasets only or learned by fusion using the Type 2 or 3 models for $K = 5$ to $K = 40$ when the true $K = 20$. The error shade indicates the standard error across 100 simulations. **(d)** The mean DCBC value for the individual maps.

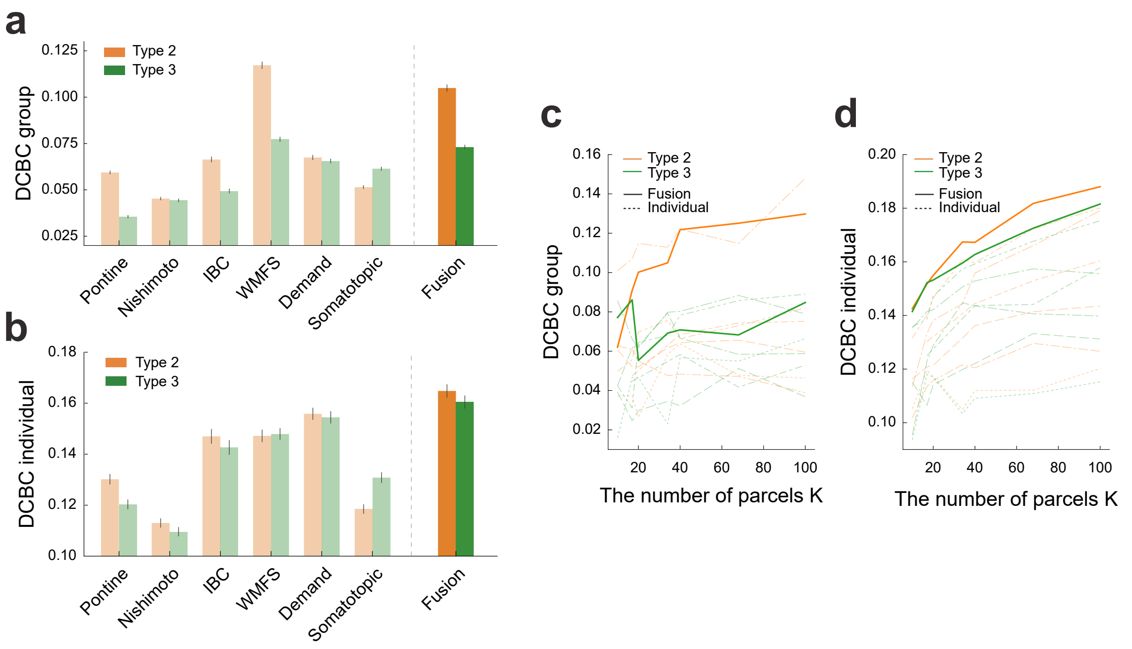

Figure S7: **Performance comparison of cerebellar parcellations learned by type 2 and 3 fusion models using 6 functional task-based datasets on resting-state data.** Mean DCBC value of the **(a)** group parcellation maps and **(b)** individual parcellation maps across subjects in the test dataset. Results are averaged across $K = 10$ to 100. **(c)** Mean DCBC value of the group map for $K = 10$ to 100. **(d)** Mean DCBC value of the individual map for $K = 10$ to 100. The test data for evaluation was the resting-state cortical-cerebellar functional connectivity profile of the HCP unrelated 100 subjects (Methods 2.2), which was calculated based on regular Icosahedron tessellation of 162 ROIs per cortical hemisphere. The resting-state connectivity profile was then calculated by correlating the raw time series at each cerebellar voxel with the averaged time courses in those cortical ROIs. The error bar reported in the figure indicates the SEM across 100 subjects.

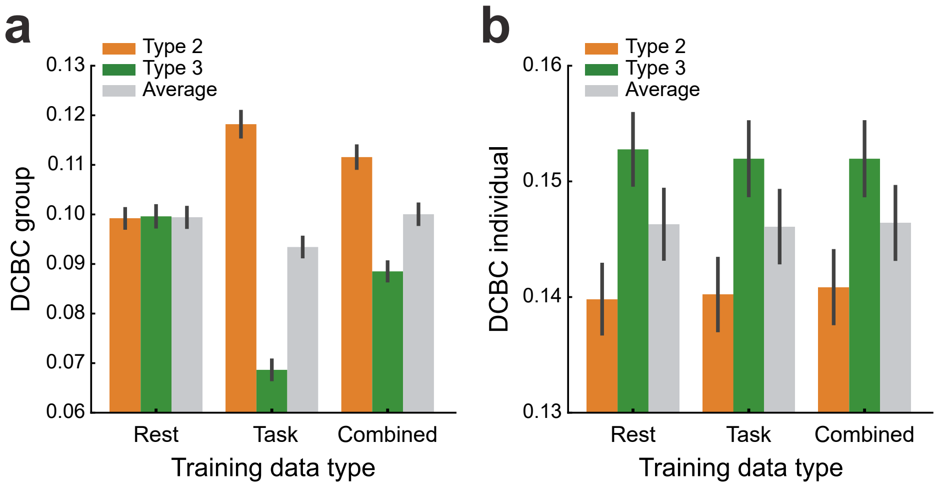

Figure S8: **Performance of cerebellar group parcellations derived from resting-state data only, task-based data only, or the combination of both on resting-state data.** Probabilistic parcellations were learned using Type 2 (orange) or 3 (green) models. The gray bar indicates the averaged performance across the two models. **(a)** Mean group DCBC, and **(b)** mean individual DCBC evaluated on the HCP left out 50 unrelated subjects. The test data for evaluation was the resting-state cortical-cerebellar functional connectivity profile of the leftover 50 HCP unrelated subjects (Results 3.6), which was calculated based on regular Icosahedron tessellation of 162 ROIs per cortical hemisphere. The resting-state connectivity profile was then calculated by correlating the raw time series at each cerebellar voxel with the averaged time courses in those cortical ROIs. The error bar reported in the figure indicates the standard error across 100 subjects. The error bar indicates the SEM across 50 subjects. Results are averaged across all tested levels of $K = 10$ to 100.

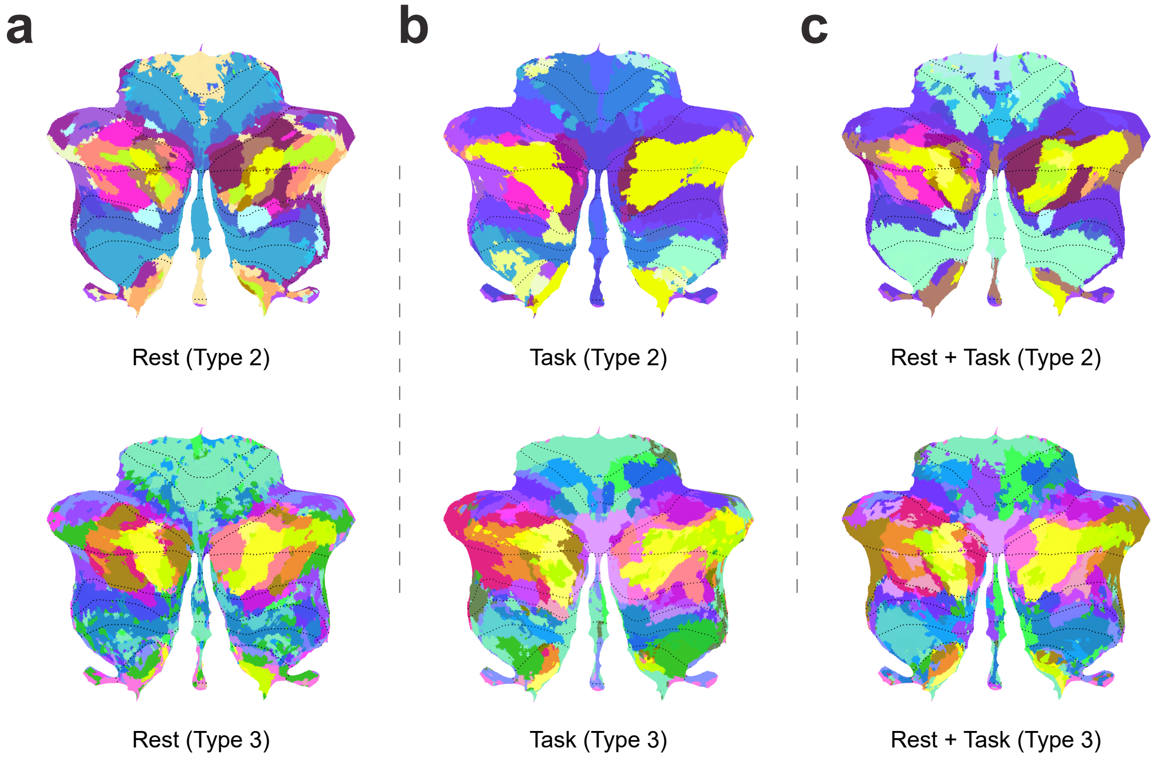

Figure S9: **The visualization of the learned group maps ($K = 34$).** **(a)** The maps were trained on a pure resting-state dataset *HCP-Unrelated 100* using the Type 2 or 3 fusion model. **(b)** The maps were purely trained on task-based datasets using the Type 2 or 3 fusion model. The task datasets are *MDTB*, *Highres-MDTB*, *Nakai&Nishimoto*, *IBC*, *WMFS*, *Demand*, *Somatotopic*. **(c)** The maps were trained on the combination of resting-state and all task-based datasets. The colors for the parcels are aligned in each type of model, where two similar colors in RGB space indicate the two parcels have similar task activation responses on average.

References

A. Banerjee, I. S. Dhillon, J. Ghosh, S. Sra, and G. Ridgeway. Clustering on the unit hypersphere using von mises-fisher distributions. *Journal of Machine Learning Research*, 6(9), 2005.

K. Hornik and B. Grün. movmf: An r package for fitting mixtures of von mises-fisher distributions. *Journal of Statistical Software*, 58(10):1–31, 2014.

F.-Y. Wu. The potts model. *Reviews of modern physics*, 54(1):235, 1982.
